# Knockdown of ribosome RNA processing protein 15 suppresses migration of hepatocellular carcinoma through inhibiting PATZ1-associated LAMC2/FAK pathway

**DOI:** 10.1186/s12885-024-12065-4

**Published:** 2024-03-12

**Authors:** Tongtong Pan, Jinhai Li, Ouyang Zhang, Yuqin Zhu, Hongfei Zhou, Mengchen Ma, Yanwen Yu, Jiaojian Lyu, Yongping Chen, Liang Xu

**Affiliations:** 1 Key Laboratory of Laboratory Medicine, Ministry of Education, School of Laboratory Medicine and Life Sciences, Wenzhou Medical University, 325035 Wenzhou, Zhejiang China; 2 Zhejiang Provincial Key Laboratory for Accurate Diagnosis and Treatment of Chronic Liver Diseases, The First Affiliated Hospital of Wenzhou Medical University, 325035 Wenzhou, Zhejiang China; 3 Department of Liver and Gall Surgery, The Third Affiliated Hospital of Wenzhou Medical University, 325200 Wenzhou, Zhejiang China; 4 Department of Infectious Diseases, Lishui People’s Hospital, 323000 Lishui, Zhejiang China

**Keywords:** Ribosome RNA processing protein 15, Hepatocellular carcinoma migration, LAMC2, Focal adhesion kinase

## Abstract

**Background:**

Ribosomal RNA processing protein 15 (RRP15) has been found to regulate the progression of hepatocellular carcinoma (HCC). Nevertheless, the extent to which it contributes to the spread of HCC cells remains uncertain. Thus, the objective of this research was to assess the biological function of RRP15 in the migration of HCC.

**Methods:**

The expression of RRP15 in HCC tissue microarray (TMA), tumor tissues and cell lines were determined. In vitro, the effects of RRP15 knockdown on the migration, invasion and adhesion ability of HCC cells were assessed by wound healing assay, transwell and adhesion assay, respectively. The effect of RRP15 knockdown on HCC migration was also evaluated in vivo in a mouse model.

**Results:**

Bioinformatics analysis showed that high expression of RRP15 was significantly associated with low survival rate of HCC. The expression level of RRP15 was strikingly upregulated in HCC tissues and cell lines compared with the corresponding controls, and TMA data also indicated that RRP15 was a pivotal prognostic factor for HCC. RRP15 knockdown in HCC cells reduced epithelial-to-mesenchymal transition (EMT) and inhibited migration in vitro and in vivo, independent of P53 expression. Mechanistically, blockade of RRP15 reduced the protein level of the transcription factor POZ/BTB and AT hook containing zinc finger 1 (PATZ1), resulting in decreased expression of the downstream genes encoding laminin 5 subunits, LAMC2 and LAMB3, eventually suppressing the integrin β4 (ITGB4)/focal adhesion kinase (FAK)/nuclear factor κB kappa-light-chain-enhancer of activated B cells (NF-κB) signaling pathway.

**Conclusions:**

RRP15 promotes HCC migration by activating the LAMC2/ITGB4/FAK pathway, providing a new target for future HCC treatment.

**Supplementary Information:**

The online version contains supplementary material available at 10.1186/s12885-024-12065-4.

## Introduction

Globally, liver cancer is the 6th most common malignancy and the 3rd leading cause of cancer-related fatalities. Hepatocellular carcinoma (HCC) stands as the prevailing form of liver cancer, with around 900,000 new cases and 820,000 fatalities recorded in 2020 alone [1]. Despite significant advances in therapeutic strategies such as surgical resection, radiofrequency ablation, liver transplantation and immunotherapy, the overall prognosis of HCC is still poor [2, 3], with the majority of patients suffering from postoperative recurrence, invasion and metastasis [4]. Extrahepatic metastases have been reported to occur in 13.5-42% of HCC cases [5], and the most common sites of blood metastasis are the lungs (up to 60% of patients with metastatic disease) and bone (up to 40% of patients) [5–7]. Hence, clarifying the specific mechanisms of cellular metastasis in HCC and finding new targets are critical to improving the survival of HCC patients.

Tumor cell interaction with the extracellular matrix (ECM) is a well-recognized link leading to tumor progression [8], which forms the scaffolding for tissues and provides structural integrity. The ECM promotes tumor metastasis through the formation of focal adhesions with the integrins on the tumor cell membrane [9, 10] via glycoproteins such as the laminins [11]. Laminin-5, encoded by the LAMA3, LAMB3 and LAMC2 genes, is the primary adhesion component of the epidermal basement membrane. Laminin 5 interacts with integrins α3β1 or α6β4 on tumor cells to activate focal adhesion kinase (FAK) and nuclear factor kappa-light-chain-enhancer of activated B cells (NF-κB), which promotes tumor cell migration and invasion [12–14].

Ribosome RNA processing protein 15 (RRP15) is a nucleolar protein required for nucleole formation [15]. A recent study showed that ribosome biogenesis is accompanied by an increase in RRP15 levels, suggesting that the latter could be a significant factor in carcinogenesis [16]. Knocking down RRP15 in cancer cells resulted in cell cycle arrest or apoptosis [15]. Deficiency of RRP15 decreased the proliferation and metastasis of colorectal cancer cells [17, 18]. Although inhibition of RRP15 has been found to inhibit HCC proliferation and growth [19], the function of RRP15 in HCC metastasis has not yet been revealed [20]. Hence, this research sought to elucidate the function of RRP15 in the migration of HCC cells, and explore the underlying mechanisms.

## Materials and methods

### Data

RNA-seq expression matrice from HCC and normal samples was obtained from GEPIA database (http://gepia.cancer-pku.cn/).

### Patients

Tumors and matched peritumoral specimens were donated by 20 patients with HCC who underwent surgical resection without preoperative treatment at Lishui People’s Hospital (Lishui, Zhejiang, China), who were informed of the objective of the study and gave informed consent. This research was granted approval by the Ethics Committee of the Lishui People’s Hospital (No. LLW-FO-401). 20 pairs of liver tissues were homogenized for total RNA extraction, and 12 pairs were used for protein isolation.

### Tissue microarrays and immunohistochemistry assay

The tissue microarray (TMA) was purchased from YEPCOME Biotechnology (YP-LVCSUR1801, Shanghai, China), consists of a total of 79 formalin-fixed and paraffin-embedded tissue sections. The TMA sections were stained with rabbit anti-RRP15 sera were applied at 1: 50 dilution [17], and the expression level of RRP15 was scored according to the signal intensity and distribution. The specific methods and scoring rules were described previously [21].

### Cell lines and cell culture

The HepG2 cells were purchased from ATCC (Washington D.C., USA), and the Huh7, MHCC-97 H, MHCC-97 L and LM3 cell lines were purchased from the Chinese Academy of Sciences’ Cell Bank (Shanghai, China). MIHA cell line was purchased from the Hunan Fenghui Biotechnology Co., Ltd (CL0469, Hunan, China). Cell lines were cultivated according to the instructions and passaged at 80% fusion.

### Small interfering RNA (siRNA) and short hairpin RNA (shRNA) transfection

SiRNAs targeting RRP15, LAMC2 and PATZ1, and the RRP15 shRNA were bought from GenePharma Company (Shanghai, China). The sequences are listed in Supplementary Table 1. ShRNA and negative control were separately cloned into pLKO.1-puro vectors (Sigma-Aldrich, Burlington, MA, USA). Full length RRP15 (NM_016052.4) cDNA was synthesized and cloned into the expression vector pCDH (Sigma-Aldrich). Cells were transfected with the respective constructs (50nM siRNAs) using Lipofectamine 3000 reagent (Invitrogen, Carlsbad, CA, USA).

### Wound healing assay

The transfected cells were cultured in six-well plates, and the monolayers were scraped longitudinally with a sterile 10-µL pipette tip once the cells were 80–90% confluent. After rinsing the cell debris with PBS, the adherent cells were cultivated in serum-free medium, and photographs of the wound area were taken at 12 h intervals over a period of 48 h to assess cell migration.

### Transwell migration and invasion assay

8 × 10^4^ cells were inoculated into the upper chamber of each well of a transwell plate (24 wells, 8 μm pore size; Corning, New York, USA) with 200 µL of serum-free medium and the lower chamber with medium containing 10% FBS. For the invasion assay, the cells were seeded in transwell filters pre-coated with 30 µL diluted (1:9) Matrigel (Corning). After incubating for 36 h, the cells remaining on the surface of the filter were removed, and those that had migrated/invaded were fixed with 4% paraformaldehyde (PFA) and stained with crystal violet (Beyotime Biotechnology, Shanghai, China). The count of migrated or invaded cells was counted in 5 random fields per well.

### Cell adhesion assay

MHCC-97 H and LM3 cells were seeded into 96-well plates pre-coated with Matrigel at the density of 8 × 10^3^ cells/well. After 1 h, the plates were rinsed with PBS, the attached cells were fixed with 4% PFA and stained with Wright’s Giemsa (Beyotime Biotechnology). The cells were photographed and counted under a microscope.

### Cell proliferation assay

MHCC-97 H and LM3 cells were inoculated into 96-well plates at a density of 2 × 10^3^ cells/well, and then transfected with different siRNAs (siNT/siRRP15-1/siRRP15-2) or treated with 5 µM Sorafenib (Solarbio Life Sciences, Beijing, China), and cultured for 24, 48, 72, 96 and 120 h respectively. 10 uL of Cell Counting Assay Kit 8 Reagent (CCK-8; Dojindo Laboratories, Kumamoto, Japan) was added to each well for incubation. Spectrophotometers (Varioskan Flash, Thermo Fisher Scientific, Waltham, MA, USA) were used to measure absorbance at 450 nm and to calculate the percentage of living cells.

### Colony formation assay

The cells were inoculated into a 6-pore panel at a 1000 cells/well. After 14 days, the colonies were washed twice with PBS, fixed with 4% PFA, stained with crystal violet (Beyotime Biotechnology), and counted.

### Apoptosis assay

MHCC-97 H and LM3 cells were inoculated in 24-well plates at a density of 2 × 10^4^ cells/well and transfected with different siRNAs (siNT/siRRP15-1/siRRP15-2). Apoptosis was assessed by flow cytometry (Becton Dickinson FACS Calibur; BD Biosciences, Franklin Lakes, NJ, USA) analysis according to the instructions of Annexin V-PE / 7-AAD Apoptosis Detection Kit (KeyGEN BioTECH, Nanjing, China).

### Cell cycle assay

MHCC-97 H and LM3 cells were seeded in 24-well plates at the density of 2 × 10^4^ cells/well, and transfected with different siRNAs (siNT/siRRP15-1/siRRP15-2). After culturing for 48 h, the cells were collected into single cells, rinsed with ice-cold PBS and fixed overnight with chilled 70% ethanol at -20℃. The cells were washed twice with PBS and incubated with 50 µg/mL propidium iodide (Solarbio Life Sciences, Beijing, China) and 0.1 mg/mL RNase A (Qiagen, Hilden, Germany) in PBS. The stained cells were analyzed in a flow cytometer.

### Establishment of in vivo tumor models

Male BALB/c nude mice (age: 5–6 weeks; weight: 18–22 g; SLAC Laboratory Animal Co. Ltd., Shanghai, China) were randomly divided into the RRP15 knockdown and control groups. The mice were injected with 3 × 10^6^ MHCC-97 H cells in 100 µL PBS and 100 µL Matrigel to create subcutaneous tumors in their right axillary fossa. The tumors were measured every 3 days using calipers, and tumor volume was calculated as (length × width^2)/2. The migration model involved the injection of 1 × 10^7^ MHCC-97 H cells in 100 µL PBS into the mice’s tail vein. Eight weeks after inoculation, mice were euthanized by CO_2_ asphyxiation, and the lungs were dissected [22]. The tissues were fixed with 4% PFA, embedded in paraffin, and stained with hematoxylin and eosin (H&E). Animal experiments were approved by the Wenzhou Medical University Animal Experiment Committee (No. wydw2022-0164).

### RNA-sequencing analysis

TRIzol™ reagent (Invitrogen) was utilized to extract total RNA from MHCC-97 H cells. LC-Bio Technology CO. Ltd. (Hangzhou, China) conducted RNA-sequencing. The entirety of the sequencing data produced in this research has been stored in the GEO database (GSE228416).

### mRNA and protein detection

Total RNA and protein were extracted from cells using TRIzol^™^ reagent (Invitrogen) and RIPA lysis buffer (Millipore, Billerica, Massachusetts, USA), respectively. Real-time PCR analyses were performed according to manufacturers’ instructions. Primers used for real-time PCR were shown in Supplementary Table S2. The expression of proteins was determined with immunoblotting. Briefly, proteins were separated by sodium dodecyl sulfate-polyacrylamide gel electrophoresis, transferred onto polyvinylidene fluoride or polyvinylidene difluoride membranes (Millipore). Then, the membrane was cut into strips and incubated overnight with primary antibodies at 4 °C (Supplementary Table 3). Blots were detected by chemiluminescence (Bio-Rad, Hercules, CA, USA) and visualized using Image Lab software (version 6.1, Bio-Rad Laboratories).

### Statistical analyses

Data were analyzed using GraphPad Prism 8 software (San Diego, CA). The experimental data were presented as the mean ± standard error of the mean (SEM) of three independent experiments performed in triplicate. Student’s *t* test and one-way analysis of variance (ANOVA) were used to compare data between groups, and *p*-value < 0.05 was considered statistically significant.

## Results

### RRP15 was upregulated in HCC tissues and cells

**Fig. 1:**
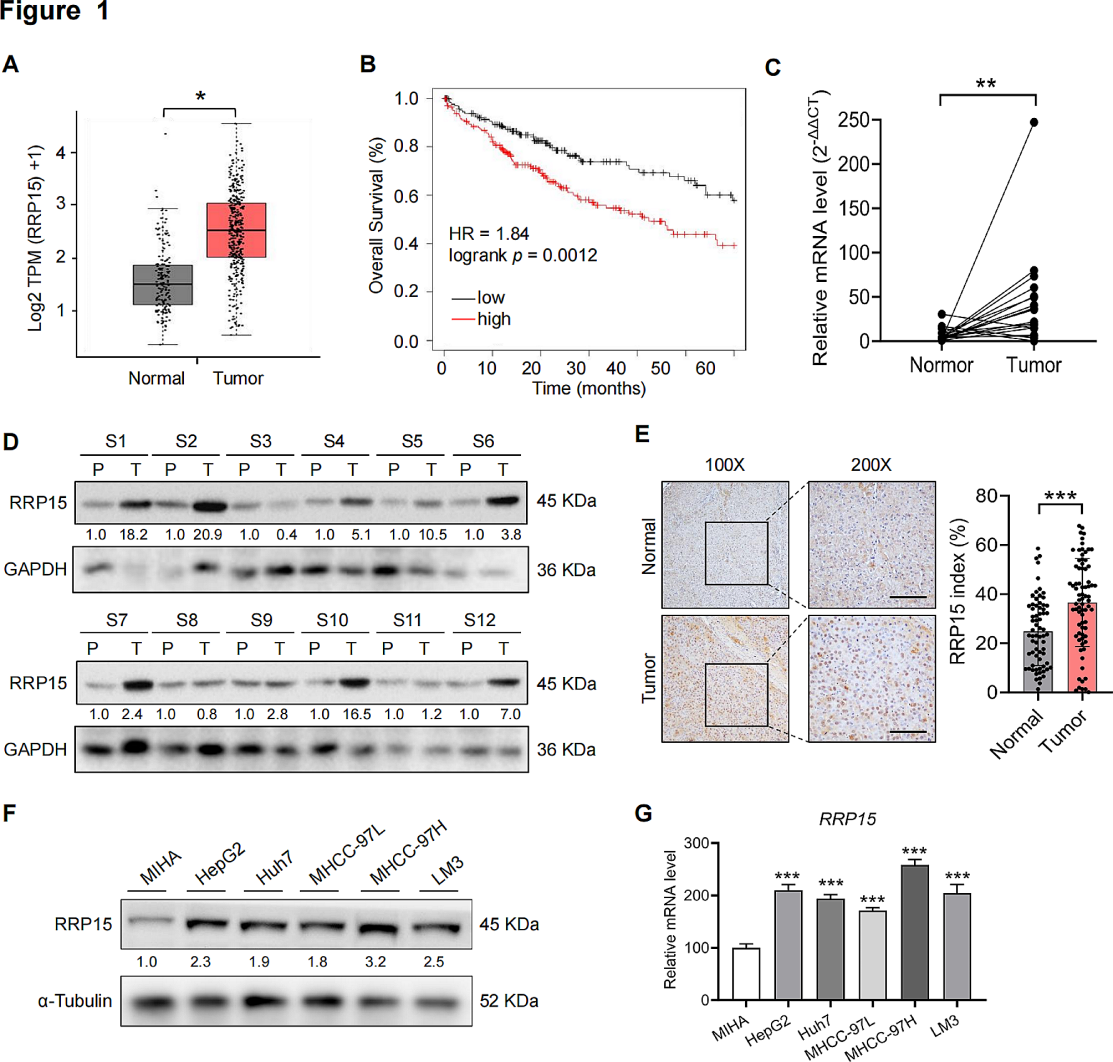
Upregulation of RRP15 in HCC tissues and cells. (**A**) The expression level of RRP15 mRNA in HCC tissues (**T**) and normal liver tissues (N) in the TCGA database. (**B**) Overall survival of HCC patients according to Kaplan-Meier database in the TCGA database. (**C**, **D**) The expression of RRP15 mRNA and protein in HCC tissues and adjacent non-tumor tissues. (**E**) Representative images and quantitative analysis of TMA stained with IHC for RRP15. Scale bar = 50 μm. (**F**, **G**) RRP15 protein and mRNA levels in five HCC cell lines and a normal liver cell line. Data are presented as mean ± SEM. *p < 0.05, **p < 0.01, ***p < 0.001. RRP15: ribosomal RNA processing protein 15; HCC: hepatocellular carcinoma; TMA: tissue microarray

RRP15 mRNA was significantly upregulated in HCC samples compared to normal liver samples in the GEPIA database (Fig. 1A). In addition, analysis of the Kaplan-Meier database revealed that high RRP15 expression was significantly associated with lower overall survival (Fig. 1B). We also evaluated RRP15 mRNA and protein levels in paired HCC and normal liver tissue samples, revealing a significant elevation in RRP15 expression within HCC tissues (Fig. 1C, D). RRP15 expression profile was accessed by IHC in a human TMA containing 79 paired HCC and peritumor tissues and representative images were shown in Fig. 1E. The results indicated that RRP15 were scored as positive expression in 36.53% of HCC tissues, as compared with 24.83% of corresponding peritumor tissues (Fig. 1E). It was further found that there was no significant difference in the protein levels of RRP15 between non-HBV-HCC patients and HBV-HCC patients (Supplementary Fig. 1A). We subsequently examined its mRNA and protein levels in five HCC cell lines (HepG2, Huh7, MHCC-97 L, MHCC97-H and LM3) as well as MIHA cells. As expected, all HCC cell lines were enriched in RRP15 compared to the control (Fig. 1F, G). In addition, we determined RRP15 expression from TCGA data based on the four major HCC etiologies, including HBV, HCV, alcoholic steatohepatitis, and non-alcoholic steatohepatitis. The results showed that the expression of RRP15 was comparable among the four HCC etiologies (Supplementary Fig. 1B). These findings suggest that RRP15 may be closely associated with HCC deterioration. In order to ascertain the involvement of RRP15 in HCC migration, we opted to conduct subsequent experiments on the metastatic MHCC-97 H and LM3 cell lines.

### RRP15 knockdown inhibited the proliferation and induced apoptosis of HCC cells

We induced gene knockdown in the MHCC-97 H and LM3 cell lines using specific siRNAs to assess the involvement of RRP15 in HCC cell progression (Supplementary Fig. 2A–B). RRP15 depletion led to a substantial decline in viability compared with that in the respective controls after 48 h of culture (Supplementary Fig. 2C). Furthermore, RRP15 knockdown resulted in a decrease of 70.4% and 36.4% in the colonies established by the MHCC-97H and LM3 cells, respectively (Supplementary Fig. 2D). Furthermore, knockdown of RRP15 markedly increased the apoptosis rates of both MHCC-97 H and LM3 cells (Supplementary Fig. 3A–B); and led to an elevated number of cells in the sub-G1 and G1 phases and a reduction in the number of cells in the G2/M phase (Supplementary Fig. 3C–D). Consistently, the lack of RRP15 upregulated P53 and attenuated PCNA, Cyclin D1 and CDK2 protein levels in both cell lines (Supplementary Fig. 3E). Collectively, the above results indicated that RRP15 knockdown suppressed the proliferation of HCC cells and attenuated tumorigenesis by inducing G1-phase arrest and apoptosis.

Additionally, in a nude mouse HCC xenograft model established using MHCC-97H cells, the tumor volume of RRP15 knockout nude mice was significantly reduced 15 days after inoculation compared to controls (Supplementary Fig. 3F). Consistent with this, RRP15 depletion also decreased the tumor weight (Supplementary Fig. 3G) and overall tumor size (Supplementary Fig. 3H) compared to that in the control group.

### RRP15 knockdown attenuated HCC migration and invasion independent of P53

**Fig. 2:**
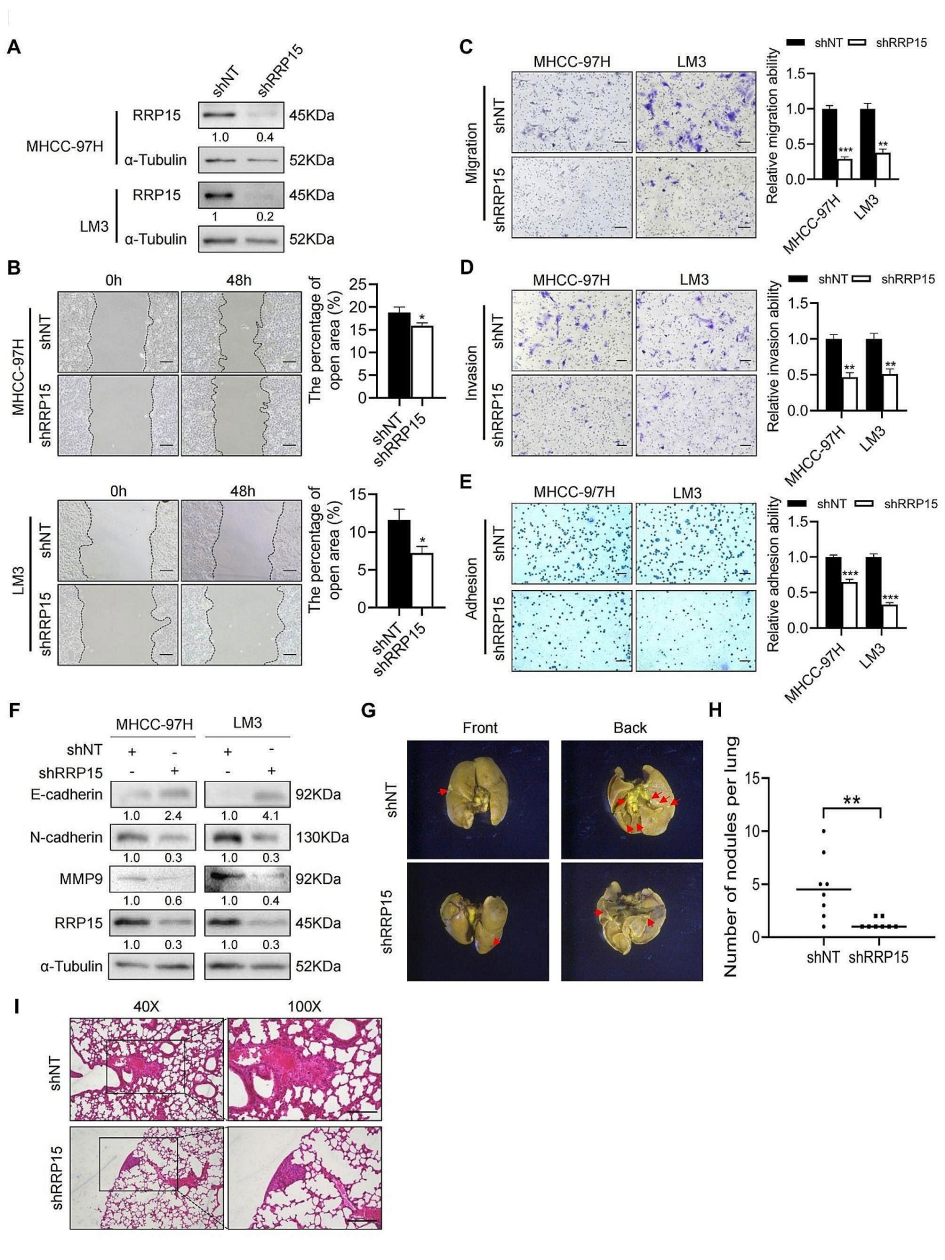
RRP15 knockdown attenuated the migration and invasion of HCC. (**A**) The knockdown efficiency of RRP15 in HCC cells. (**B**) Downregulation of RRP15 inhibited migration of HCC cell in the wound healing assay. Scale bars = 100 μm. (**C**-**D**) Migration and invasion of MHCC-97 H or LM3 cells transfected with shNT or shRRP15 in the transwell assay. Scale bars = 100 μm. (**E**) Downregulation of RRP15 inhibited adhesion of HCC cells. Scale bars = 100 μm. (**F**) Expression levels of EMT-related proteins in MHCC-97 H or LM3 cells transfected with shNT or shRRP15. (**G**) Representative images of lung metastases observed under a stereoscope. Scale bars = 1 cm. (**H**) Number of metastatic nodules in the lung tissues in the indicated groups (*n* = 8 per group). (**I**) Representative images of H&E-stained lung tissue sections in shNT/shRRP15 mice. Scale bars = 100 μm. Data are presented as mean ± SEM. *p < 0.05, **p < 0.01, ***p < 0.001. RRP15: ribosomal RNA processing protein 15; HCC: hepatocellular carcinoma. H&E: hematoxylin and eosin

The effect of RRP15 on HCC migration was also ascertained by shRNA-mediated knockdown in MHCC-97H and LM3 cells (Fig. 2A). And in the wound healing assay, inhibition of RRP15 remarkably reduced the migration of HCC cells (Fig. 2B). Furthermore, knocking down RRP15 markedly suppressed the migration and invasion of MHCC-97 H and LM3 cells in the transwell assay (Fig. 2C–D), and decreased the adhesion of HCC cells (Fig. 2E). EMT is a pivotal process of HCC migration [23], unsurprisingly, RRP15 knockdown promoted the expression of epithelial marker E-cadherin, and diminished the mesenchymal markers N-cadherin and matrix metallopeptidase (MMP) 9 in the HCC cell lines (Fig. 2F).

Since knockdown of RRP15 increased P53 expression, we hypothesized that RRP15 regulation of HCC migration dependent on P53. Thus, we used siRNA to block the expression of RRP15 in P53-deficient cell line Hep3B (Supplementary Fig. 4A–B). The data revealed knockdown of RRP15 could also attenuate the migration, invasion, and adhesion of the Hep3B cells (Supplementary Fig. 4C–E), indicating that the regulation of RRP15 knockdown on the migration of HCC did not depend on the increase of P53 expression.

On the other hand, in a mouse lung migration model constructed by intravenously injecting MHCC-97 H cells, the number of lung metastatic nodules was obviously increased in mice injected with shNT cells compared with those injected with shRRP15 cells (Fig. 2G, H), and aggressive tumor areas in the lung sections (Fig. 2I). In summary, knockdown of RRP15 attenuated HCC migration.

#### Inhibition of RRP15 downregulated the focal adhesion pathway

**Fig. 3:**
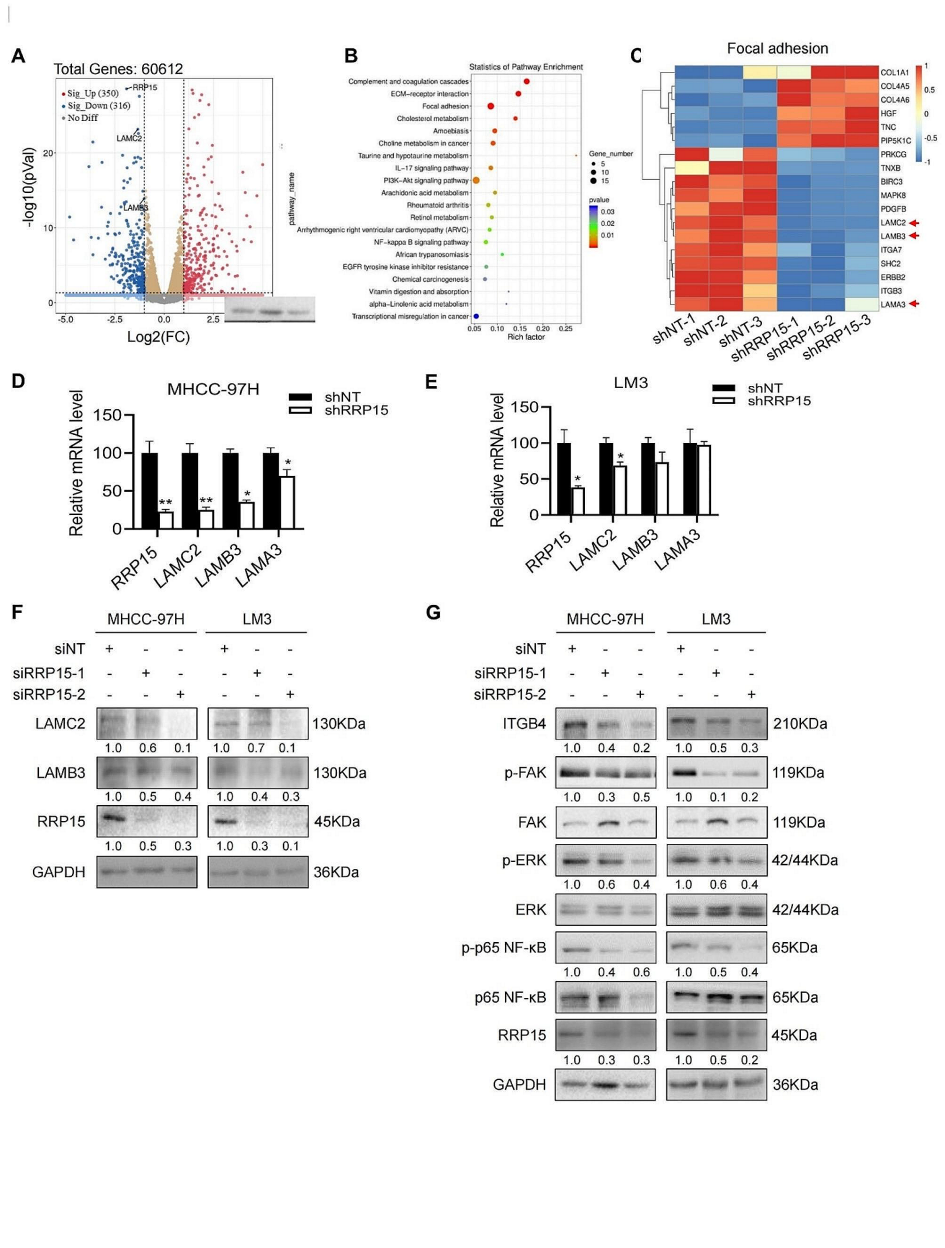
RRP15 knockdown inactivated the LAMC2/ITGB4/FAK pathway in HCC cells. (**A**) The volcano plot of differentially expressed genes (DEGs). The blue dots represent downregulated genes, the grey dots are non-differentially expressed genes and the red dots are upregulated genes. (**B**) KEGG pathway analysis showing the significantly associated signaling pathways in the RRP15-knockdown MHCC-97 H cells. (**C**) Heatmap of DEGs. Up- and down-regulated genes are shown in red and blue respectively. (**D**-**E**) Effect of RRP15 knockdown on the expression of LAMC2, LAMB3 and LAMA3 mRNAs. (**F**) Effect of RRP15 knockdown on the expression of LAMC2 and LAMB3 proteins. (**G**) Immunoblots showing ITGB4, pFAK, pERK, p-p65, FAK, ERK and p65 levels in RRP15-knockdown and control cells. Data are presented as mean ± SEM. *p < 0.05, **p < 0.01. RRP15: ribosomal RNA processing protein 15; LAMC2: laminin subunit gamma 2; ITGB4: integrin subunit beta 4; FAK: focal adhesion kinase; HCC: hepatocellular carcinoma; LAMB3: laminin subunit beta 3; ERK: mitogen-activated protein kinase 1

To further investigate the mechanisms underlying RRP15-mediated regulation of HCC migration, we analyzed the transcriptomic changes in MHCC-97H cells with a stable knockdown of RRP15. RNA sequencing revealed that compared to the control cells, the RRP15-knockdown cells had 350 up-regulated genes and 316 down-regulated genes were by two-fold (Fig. 3A). Moreover, Kyoto Encyclopedia of Genes and Genomes (KEGG) enrichment analysis showed that the differentially expressed genes were mainly associated with focal adhesion, complement and coagulation cascades, and ECM-receptor interaction (Fig. 3B). The focal adhesion pathway consists of multiple genes involved in cell motility and cancer metastasis [24], suggesting that RRP15 knockdown regulates the migration and invasion of HCC cells. Further screening for genes involved in focal adhesion and ECM-receptor interaction, we found that *COL1A1, COL4A5, COL4A6, HGF, TNC and PIP5K1C* genes were upregulated, whereas *PRKCG, TNXB, BIRC3, MAPK8, PDGFB, LAMC2, LAMB3, ITGA7, SHC2, ERBB2, ITGB3* and *LAMA3* were downregulated (Fig. 3C). *LAMA3, LAMC2* and *LAMB3* respectively encode for the α1, γ2 and β3 chains of laminin-5 [25], which is a key factor involved in focal adhesion. RRP15 knockdown decreased LAMC2, LAMB3 and LAMA3 mRNA levels in the MHCC-97 H cells, and downregulated LAMC2 and LAMB3 in LM3 cells (Fig. 3D, E). In addition, RRP15 depletion also reduced LAMC2 and LAMB3 protein levels in both cell lines (Fig. 3F). Laminin-5 is the main ligand of integrin α6β4, and the binding of integrin β4 to LAMC2 facilitates tumor metastasis through FAK [26]. Consistent with this, knockdown of RRP15 decreased ITGB4, p-FAK, p-ERK and p-p65 NF-κB levels in the HCC cells (Fig. 3G).

### RRP15 enhanced migration of HCC cells through the LAMC2/ITGB4/FAK pathway

**Fig. 4:**
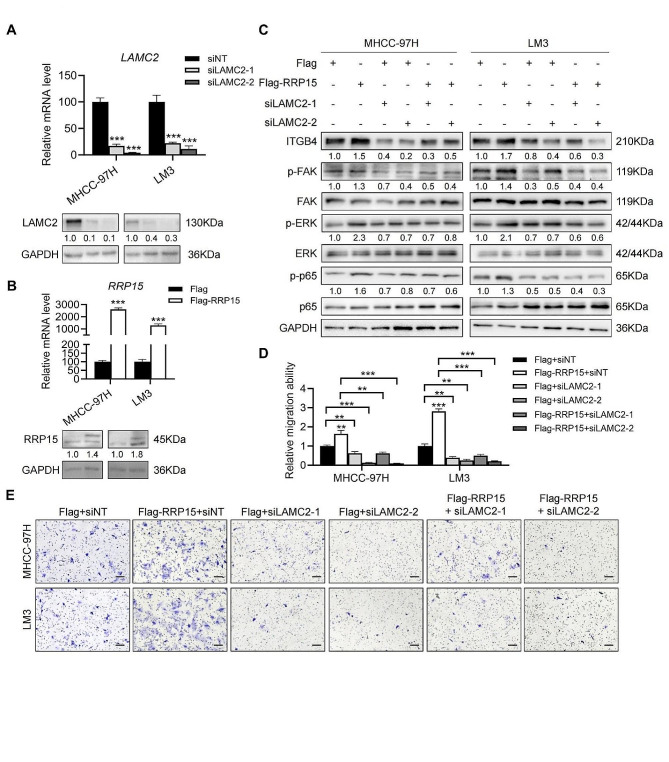
RRP15 enhanced migration of HCC cells through the LAMC2/ITGB4/FAK pathway. (**A**) The knockdown efficiency of LAMC2 in HCC cells. (**B**) The overexpression of RRP15 in HCC cells. (**C**) Expression levels of the indicated proteins in RRP15-overexpressing MHCC-97 H and LM3 cells with or without LAMC2 knockdown. (**D**-**E**) Migration and invasion of RRP15-overexpressing MHCC-97 H and LM3 cells with or without LAMC2 knockdown in the transwell assay. Scale bars = 100 μm. Data are presented as mean ± SEM. *p < 0.05, **p < 0.01, ***p < 0.001. RRP15: ribosomal RNA processing protein 15; LAMC2: laminin subunit gamma 2; ITGB4: integrin subunit beta 4; FAK: focal adhesion kinase; HCC: hepatocellular carcinoma

To further determine whether RRP15 knockdown suppressed HCC migration in a LAMC2-dependent manner, we simultaneously overexpressed RRP15 and knocked down LAMC2 in both cell lines (Fig. 4A, B), and found that overexpression of RRP15 increased ITGB4 protein levels, and those of phosphorylated FAK, ERK and p65 NF-κB. Furthermore, knocking down LAMC2 decreased FAK and p65 NF-κB phosphorylation and downregulated ITGB4 in the RRP15-overexpressing HCC cells (Fig. 4C), but did not restore the levels of p-ERK. LAMC2 knockdown also inhibited the migration of RRP15-overexpressing cells (Fig. 4D–E). Taken together, RRP15 knockdown suppressed HCC migration by inhibiting the ITGB4/FAK/NF-κB signaling pathway through LAMC2.

#### RRP15 promoted the transcription of LAMC2 and LAMB3 through PATZ1 in HCC cells

**Fig. 5:**
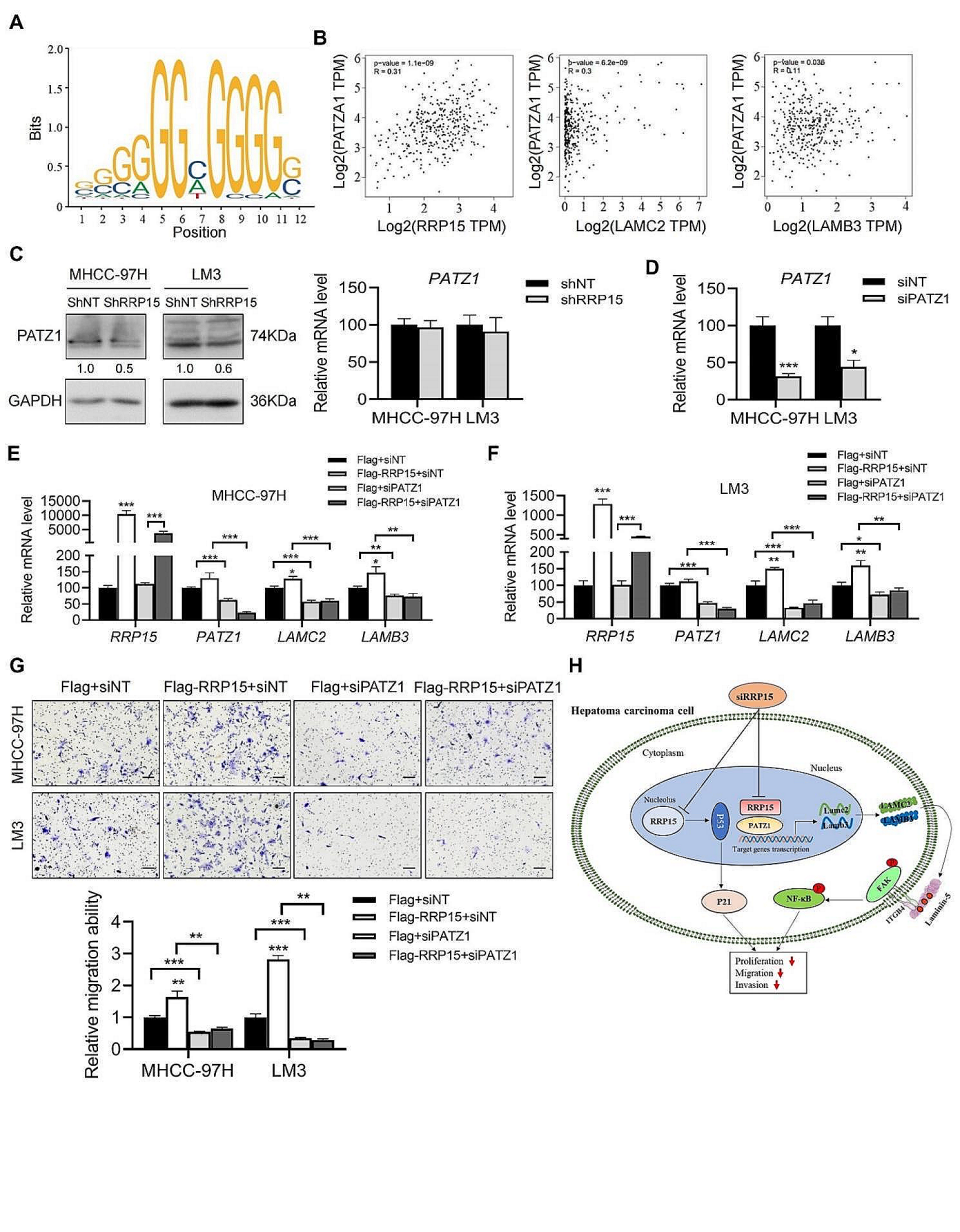
Knockdown PATZ1 suppressed LAMC2 and LAMB3 transcription in HCC cells. (**A**) Transcriptional binding site of PATZ1. (**B**) Correlation of PATZ1 mRNA level with RRP15, LAMC2 and LAMB3 mRNA levels. (**C**) Protein and mRNA levels of PATZ1 in RRP15 knockdown cells. (**D**) The knockdown efficiency of PATZ1 in HCC cells. (**E**-**F**) Expression levels of the indicated mRNAs in RRP15-overexpressing MHCC-97 H and LM3 cells with or without PATZ1 knockdown. (**G**) Migration and invasion of RRP15-overexpressing MHCC-97 H and LM3 cells with or without PATZ1 knockdown in the transwell assay. Scale bars = 100 μm. (**H**) Schematic diagram showing that knockdown of RRP15 inhibits the growth and metastasis of HCC cells via inactivation of the LAMC2 /FAK signaling. Data are presented as means ± SEM. *p < 0.05, **p < 0.01, ***p < 0.001. PATZ1: POZ/BTB and AT hook containing zinc finger 1; LAMC2: laminin subunit gamma 2; LAMB3: laminin subunit beta 3; HCC: hepatocellular carcinoma

Given that RRP15 is mainly localized in the nucleus [15], it is possible that it acts as a transcription factor in the nucleus to regulate LAMC2 and LAMB3 gene expression. We identified the POZ/BTB and AT hook containing zinc finger 1 (PATZ1) in the common promoter region of LAMC2 and LAMB3 by JASPAR database analysis (Fig. 5A). PATZ1 expression was positively correlated with that of RRP15, LAMC2 and LAMB3 in the TCGA database (Fig. 5B). Furthermore, despite comparable mRNA levels of PATZ1, stable knockdown of RRP15 in both cell lines decreased PATZ1 expression (Fig. 5C). Therefore, we stably knocked down PATZ1 in the MHCC-97 H and LM3 cells to explore its effect on RRP15-dependent regulation of HCC migration (Fig. 5D). While overexpressing RRP15 increased the expression of LAMC2 and LAMB3 mRNAs, knocking down PATZ1 led to their downregulation in the RRP15-overexpressing HCC cells (Fig. 5E–F). Consistently, PATZ1 knockdown prevented the migration of RRP15-overexpressing HCC cells (Fig. 5G). Overall, our data suggest that RRP15 promotes HCC migration via PATZ1-mediated transcriptional regulation of LAMC2 and LAMB3.

## Discussion

Zhao et al. showed that ablation of RRP15 prevented HCC proliferation and growth both in a p53-dependent manner in vitro and in vivo [19]. However, whether and how RRP15 affects the migration of HCC has not been studied so far. Since the high migration rates are responsible for the high mortality rate of patients with HCC [5–7], it is momentous to unearth new molecular markers that can accurately predict HCC migration. We innovatively found that high expression of RRP15 was associated with lower survival in HCC patients. Knocking down RRP15 expression prevented the proliferation, migration and invasion of HCC cells, independent of P53 expression, and also suppressed lung migration in a mouse model.

Laminin-5 is considerably associated with the growth, metastasis and prognosis of HCC tumors [27, 28]. It consists of the laminin α3, γ2 and β3 subunits, which are encoded by *LAMA3*, *LAMC2* and *LAMB3* respectively [25]. LAMC2 is overexpressed in multiple cancers and drives tumorigenesis by interacting with α6β4 and α3β1 integrins, epidermal growth factor receptor (EGFR), and other surface receptors [26, 27, 29–32]. In addition, deletion of LAMC2 suppressed the EMT and lymph node metastasis of cholangiocarcinoma via inactivation of the EGFR signaling pathway [33, 34]. LAMC2 is also overexpressed in ovarian cancer and can regulate tumor cell proliferation and metastasis [35]. We similarly found that RRP15 overexpression promoted the migration of HCC cells in vitro, and its oncogenic effect was abrogated by the simultaneous knockdown of LAMC2. Furthermore, the lack of RRP15 also decreased the expression of LAMC2 in HCC cells.

LAMC2 binds to integrins α6β4 and phosphorylates FAK, and activates downstream signaling pathways, such as those already reported in esophageal squamous cell carcinoma and breast cancer [26, 36–38]. FAK protein overexpression is highly associated with aggressive behavior and undesirable outcome in HCC [39, 40]. In colon cancer cells, FAK phosphorylation regulates E-cadherin expression by activating the Src signaling pathways [41]. Our results indicate that knockdown of RRP15 inhibited the FAK signaling pathway, whereas overexpression increased the level of phosphorylated FAK. LAMC2 knockdown attenuated FAK signaling in the RRP15-overexpressing cells. Furthermore, NF-κB, a downstream effector of FAK [42, 43], was also downregulated by RRP15 knockdown. Altogether, these findings suggest that knockdown of RRP15 suppresses HCC migration via attenuation of LAMC2/FAK/NF-κB signaling.

In addition, we also found an interesting phenomenon that loss of RRP15 up-regulates the expression of fibrogenic genes, including *COL1A1*, *COL4A5* and *COL4A6*, suggesting that RRP15 may inhibit fibrosis. The role of tumor-associated fibrosis in cancer progression is inconclusive, and increasing evidence has revealed that tumor-associated fibrosis inhibits the development, proliferation and metastasis of cancer. Alkasalias et al. found that fibroblast fusion into monolayers effectively inhibited tumor cell proliferation in vitro [44]. Mechanistically, hedgehog, as a key signaling pathway that promotes fibrosis, may play an important role in stromal cells inhibiting tumor progression. For example, genetic and pharmacological inhibition of hedgehog accelerates the progression of pancreatic and bladder cancers [45, 46]; hedgehog agonists induce stromal hyperplasia but reduce epithelial cell proliferation, thereby inhibiting cancer development [45]. However, the role of RRP15 in fibrosis has not been reported, and we speculate that RRP15 may also promote the progression of HCC by inhibiting fibrosis, but this speculation and the underlying mechanism need to be further studied.

Further, the predominantly nuclear location of RRP15 suggests that it may regulate gene expression [15]. Dong et al. showed that RRP15 promotes colorectal cancer metastasis through regulating leucine zipper tumor suppressor 2-mediated β-catenin signaling [17]. We found that RRP15 regulates downstream target genes at the transcriptional level, and RRP15 depletion attenuated LAMC2 expression, at least in part, through the inactivation of PAZT1 promotor. Nevertheless, we were unable to conclusively identify the mechanisms through which RRP15 regulates LAMC2, and the purported interaction between RRP15 and the transcription factor PATZ1 needs to be investigated further.

## Conclusions

RRP15, a potential HCC biomarker and therapeutic target, upregulates LAMC2 through the transcription factor PATZ1, which promotes the migration of HCC cells (Fig. 5H), whereas knockdown of RRP15 inhibits the migration of HCC by attenuating LAMC2/integrin β4/FAK signaling.

### Electronic supplementary material

Below is the link to the electronic supplementary material.

Supplementary Material 1

Supplementary Material 2

## Data Availability

The dataset supporting the conclusions of this article is available in the [GSE228416]. All data are available upon request from the corresponding author.
