# Supplementary material for: Knockdown of ribosome RNA processing protein 15 suppresses migration of hepatocellular carcinoma through inhibiting PATZ1-associated LAMC2/FAK pathway
Source: BMC Cancer. 2024 Mar 12;24:334. doi: 10.1186/s12885-024-12065-4 (PMC10936014; doi:10.1186/s12885-024-12065-4)
Supplement: Supplementary file 1 — Supplementary Material 1 [file 12885_2024_12065_MOESM1_ESM.docx]

**Title: Knockdown of ribosome RNA processing protein 15 suppresses migration of hepatocellular carcinoma through inhibiting PATZ1-associated LAMC2/FAK pathway**

Tongtong Pan^2,#^, Jinhai Li^3,#^, Ouyang Zhang^1^, Yuqin Zhu^1^, Hongfei Zhou^2^, Mengchen Ma^1^, Yanwen Yu^1^, Jiaojian Lyu^4^, Yongping Chen^2,^*, Liang Xu^1,^*

^1^ Key Laboratory of Laboratory Medicine, Ministry of Education, School of Laboratory Medicine and Life Sciences, Wenzhou Medical University, Wenzhou 325035, Zhejiang, China.

^2^ Zhejiang Provincial Key Laboratory for Accurate Diagnosis and Treatment of Chronic Liver Diseases, The First Affiliated Hospital of Wenzhou Medical University, Wenzhou 325035, Zhejiang, China.

^3^ Department of Liver and Gall Surgery, The Third Affiliated Hospital of Wenzhou Medical University, Wenzhou 325200, Zhejiang, China

^4^ Department of Infectious Diseases, Lishui People's Hospital, Lishui 323000, Zhejiang, China.

^#^ These authors contributed equally to this work.

**Supplementary Tables:**

**Table S1. siRNA and shRNA sequences.**

| **siRNA/shRNA** | **Organism** | **5'- Sequence -3'** |
| --- | --- | --- |
| siRRP15-1 | human | AAATGGTAACTGGAGCCGTA |
| siRRP15-2/shRRP15 | human | CCTGAAAGTAAACCTACTATT |
| siLAMC2-1 | human | GTCAAAGCCTGTCCTTTGA |
| siLAMC2-2 | human | TCGGGAACTTCACAGACAA |
| siPATZ1 | human | AACCGACCTCATCAGCAGGAACTTG |

**Table S2. Primers used for RT-qPCR.**

| **Primer** | **Organism** | **5'- Sequence -3'** |
| --- | --- | --- |
| GAPDH-F | human | TTCATTGACCTCAACTACATGGTTTAC |
| GAPDH-R | human | TGACAAGCTTCCCGTTCTCA |
| RRP15-F | human | GGTAACTGGAGCCGTAG |
| RRP15-R | human | GGACTTTAGCCATAGCAT |
| LAMC2-F | human | GCCTTTTGGCACCTGTATTC |
| LAMC2-R | human | CAGGATTCTCATCCCCTGAA |
| LAMB3-F | human | CCAAAGGTGCGACTGCAATG |
| LAMB3-R | human | AGTTCTTGCCTTCGGTGTGG |
| LAMA3-F | human | AAAGCGTATGTGGATAAATGTGG |
| LAMA3-F | human | CGGAAAGCAGGCGTAGAAA |
| PATZ1-F | human | ACTTGGGCTTCCCTTTGG |
| PATZ1-R | human | GCACTGGATGCCACACTG |

Table S3. Western blotting antibody information

| **Protein** | **Source** | **Catalog Number** | **Dilution** |
| --- | --- | --- | --- |
| RRP15 | Abcam, Massachusetts, USA | ab121832 | 1:1000 |
| LAMC2 | Abcam, Massachusetts, USA | ab210959 | 1:1000 |
| LAMB3 | Abcam, Massachusetts, USA | ab97765 | 1:1000 |
| α-Tubulin | Cell Signaling Technology, Massachusetts, USA | 3873S | 1:1000 |
| GAPDH | Cell Signaling Technology, Massachusetts, USA | 5174 | 1:1000 |
| Integrin β4 | Cell Signaling Technology, Massachusetts, USA | 14803 | 1:1000 |
| p-FAK (Y397) | Cell Signaling Technology, Massachusetts, USA | 8556 | 1:1000 |
| FAK | Cell Signaling Technology, Massachusetts, USA | 3285 | 1:1000 |
| p-ERK1/2 (Thr202/Tyr204) | Cell Signaling Technology, Massachusetts, USA | 9101 | 1:1000 |
| ERK | Cell Signaling Technology, Massachusetts, USA | 9102 | 1:1000 |
| p-p65 (Ser536) | Cell Signaling Technology, Massachusetts, USA | 3033 | 1:1000 |
| p65 | Cell Signaling Technology, Massachusetts, USA | 8242 | 1:1000 |
| E-cadherin | Proteintech, Wuhan, China | 20874-1-AP | 1:50000 |
| N-cadherin | Proteintech, Wuhan, China | 22018-1-AP | 1:5000 |
| MMP9 | Proteintech, Wuhan, China | 10375-2-AP | 1:1000 |
| p53 | Proteintech, Wuhan, China | 10442-1-AP | 1:10000 |
| PCNA | Proteintech, Wuhan, China | 10205-2-AP | 1:10000 |
| Cyclin D1 | Proteintech, Wuhan, China | 60186-1-Ig | 1:10000 |
| CDK2 | Proteintech, Wuhan, China | 10122-1-AP | 1:1000 |

**Supplementary Figures:**

**
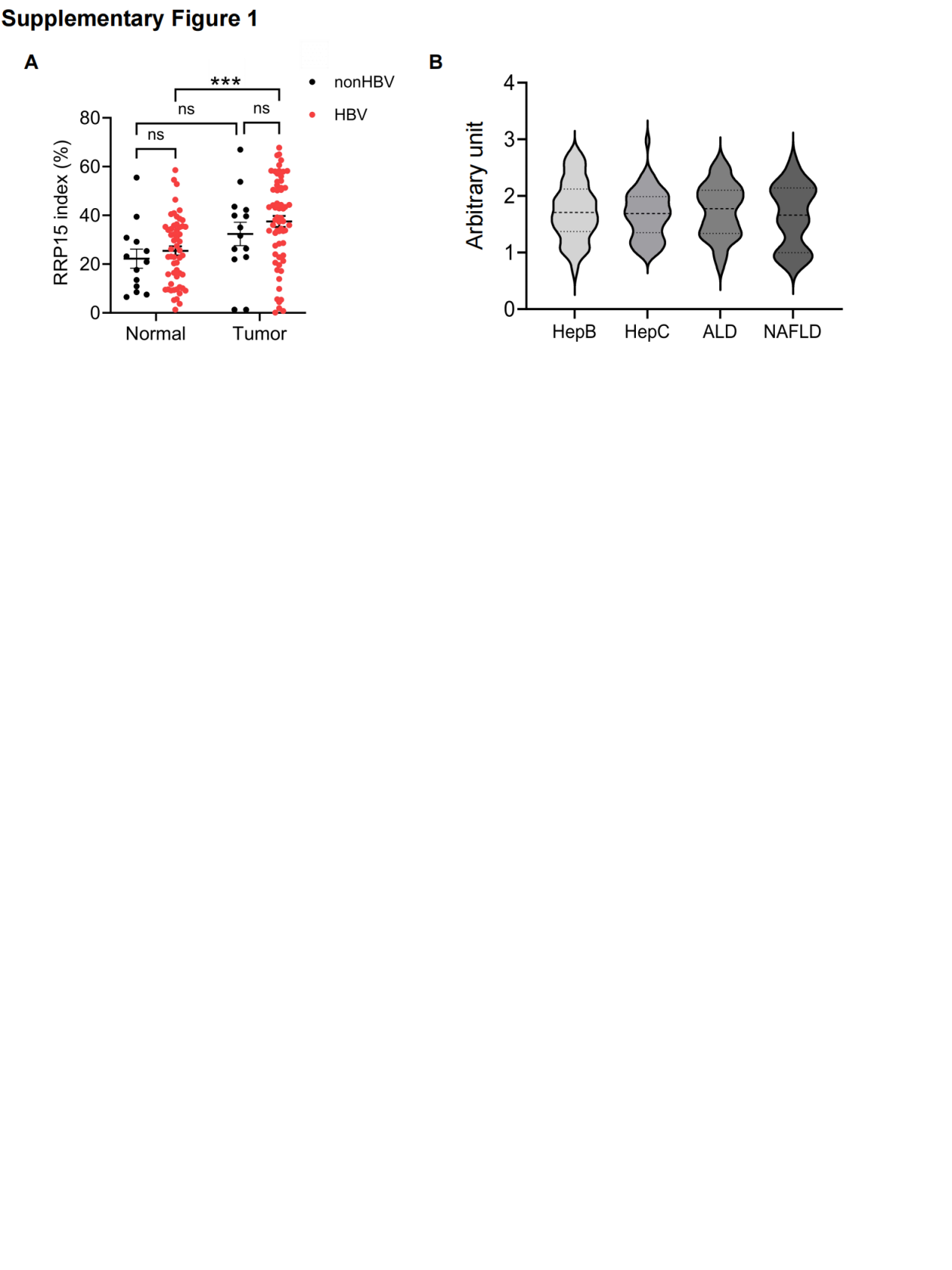
**

**Supplementary Figure 1.** RRP15 expression was comparable among the major HCC etiologies. (A) RRP15 protein levels in non-HBV-HCC patients and HBV-HCC patients in TMA samples. (B) The RRP15 expression of TCGA data. Data are presented as means ± SEM. *p < 0.05, **p < 0.01, ***p < 0.001.

**
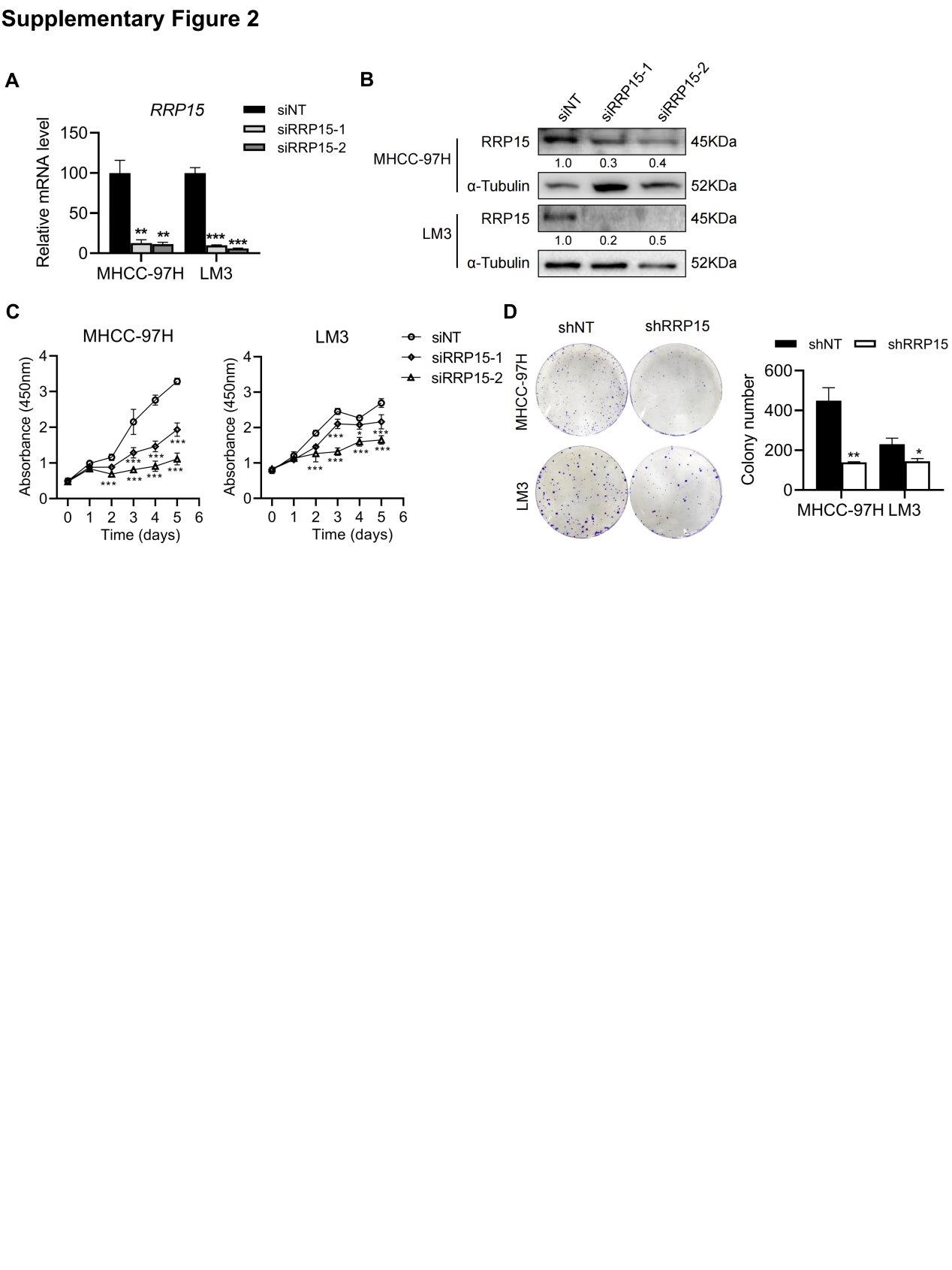
**

**Supplementary Figure 2.** Knockdown of RRP15 decreased the proliferation of HCC cells. (A-B) The knockdown efficiency of RRP15 in HCC cells. (C) CCK8 assays for HCC cell proliferation after knockdown of RRP15. (D) Colony formation assay to detect cell proliferation after knockdown of RRP15. Data are presented as means ± SEM. *p < 0.05, **p < 0.01, ***p < 0.001.


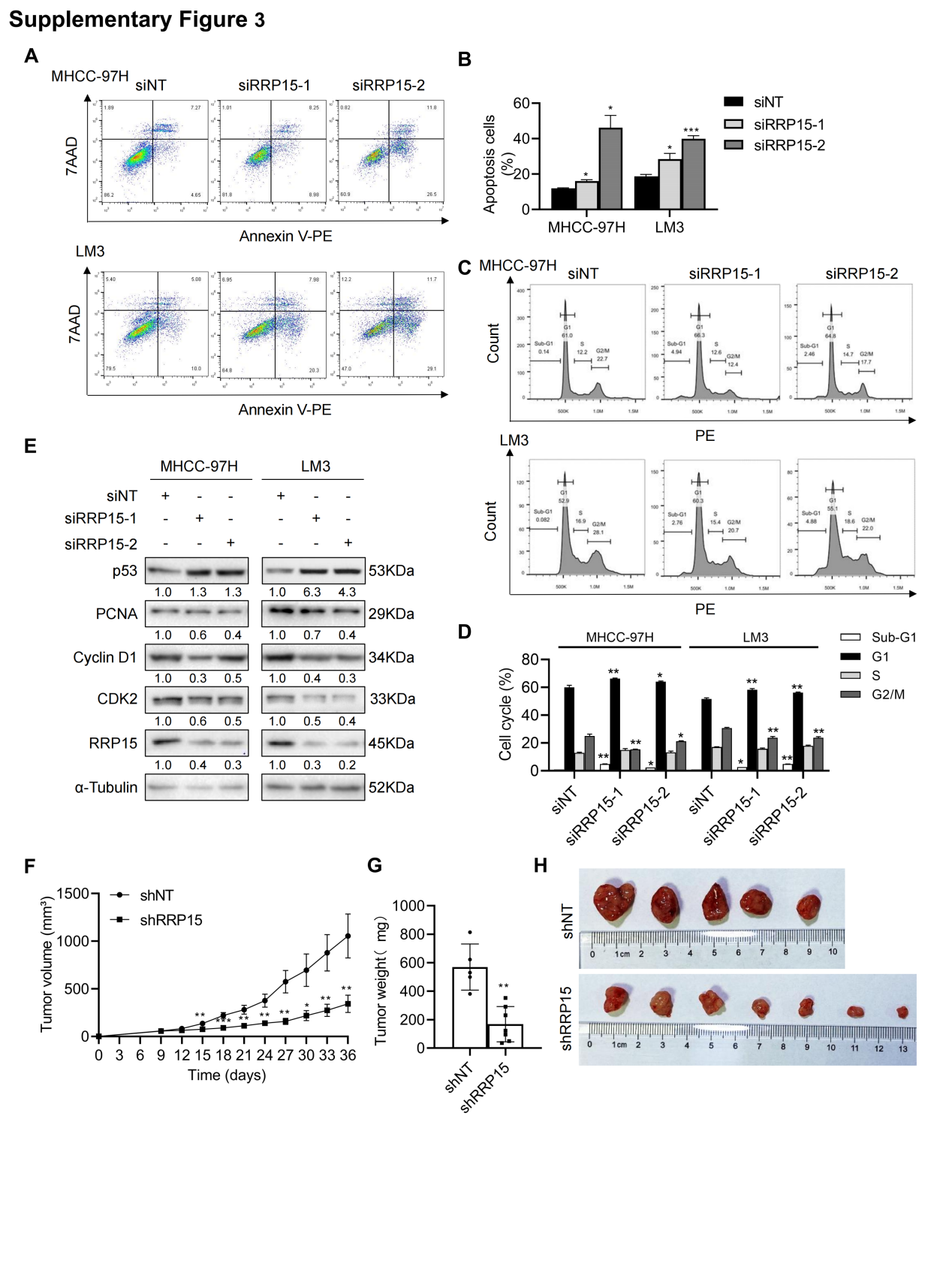


**Supplementary Figure 3.** Knockdown of RRP15 caused apoptosis and cell cycle arrest in HCC cells. (A, B) Apoptotic rate of HCC cells after RRP15 knockdown. (C, D) Flow cytometry detection of cell cycle after RRP15 knockdown. (E) Immunoblots of proliferation and cycle-related proteins after RRP15 knockdown. (F) Growth curve of subcutaneous tumor in nude mice, V=1/2×a×b2 (a is the long axis, b is the short axis). (G) Transplant tumor weight. (H) Tumor images. Data are presented as means ± SEM. *p < 0.05, **p < 0.01, ***p < 0.001.


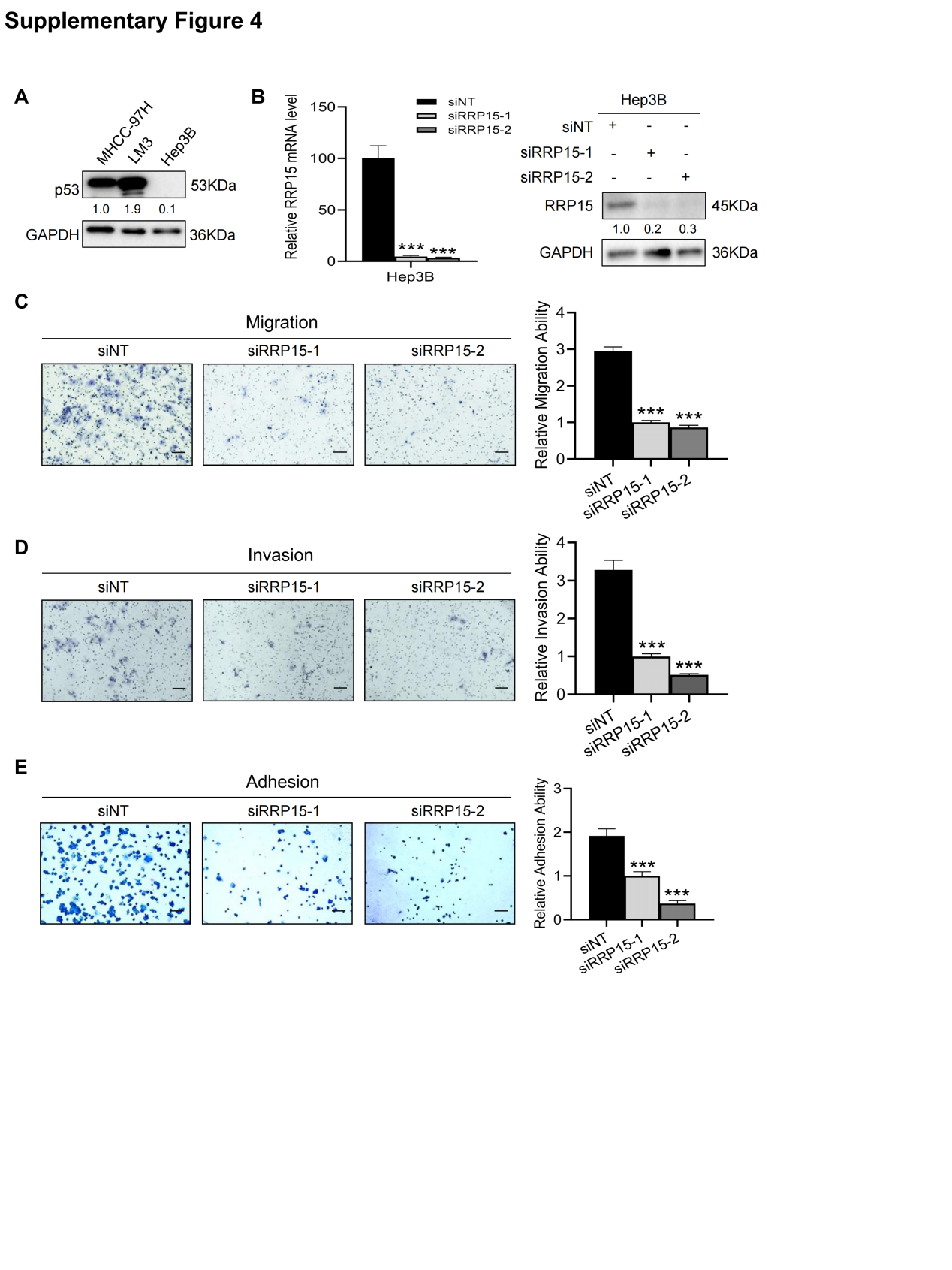


**Supplementary Figure 4.** RRP15 knockdown attenuated HCC migration independent on the expression of P53. (A) Immunoblots of P53 in HCC cells. (B) Knockdown of RRP15 in Hep3B cells. (C-E) The migration, invasion, and adhesion of HCC cells. Scale bars = 100 µm. Data are presented as mean ± SEM. **p* < 0.05, ***p* < 0.01, ****p* < 0.001.
