# Supplementary material for: Knockdown of ribosome RNA processing protein 15 suppresses migration of hepatocellular carcinoma through inhibiting PATZ1-associated LAMC2/FAK pathway
Source: BMC Cancer. 2024 Mar 12;24:334. doi: 10.1186/s12885-024-12065-4 (PMC10936014; doi:10.1186/s12885-024-12065-4)
Supplement: Supplementary file 2 — Supplementary Material 2 [file 12885_2024_12065_MOESM2_ESM.pptx]

## Slide 1
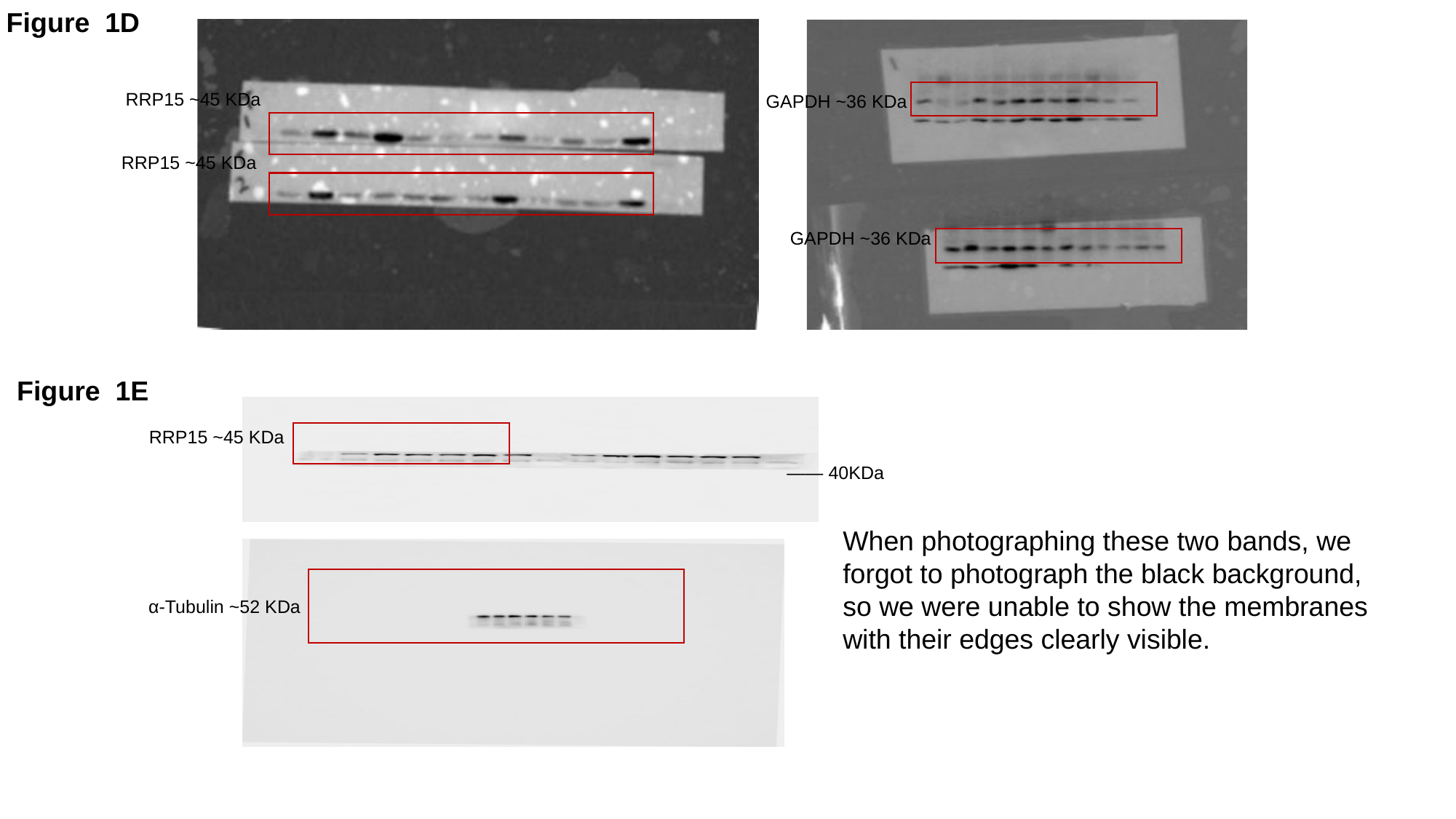

Figure 1D
RRP15 ~45 KDa
GAPDH ~36 KDa
RRP15 ~45 KDa
GAPDH ~36 KDa
Figure 1E
RRP15 ~45 KDa
—— 40KDa
When photographing these two bands, we forgot to photograph the black background, so we were unable to show the membranes with their edges clearly visible.
α-Tubulin ~52 KDa

## Slide 2
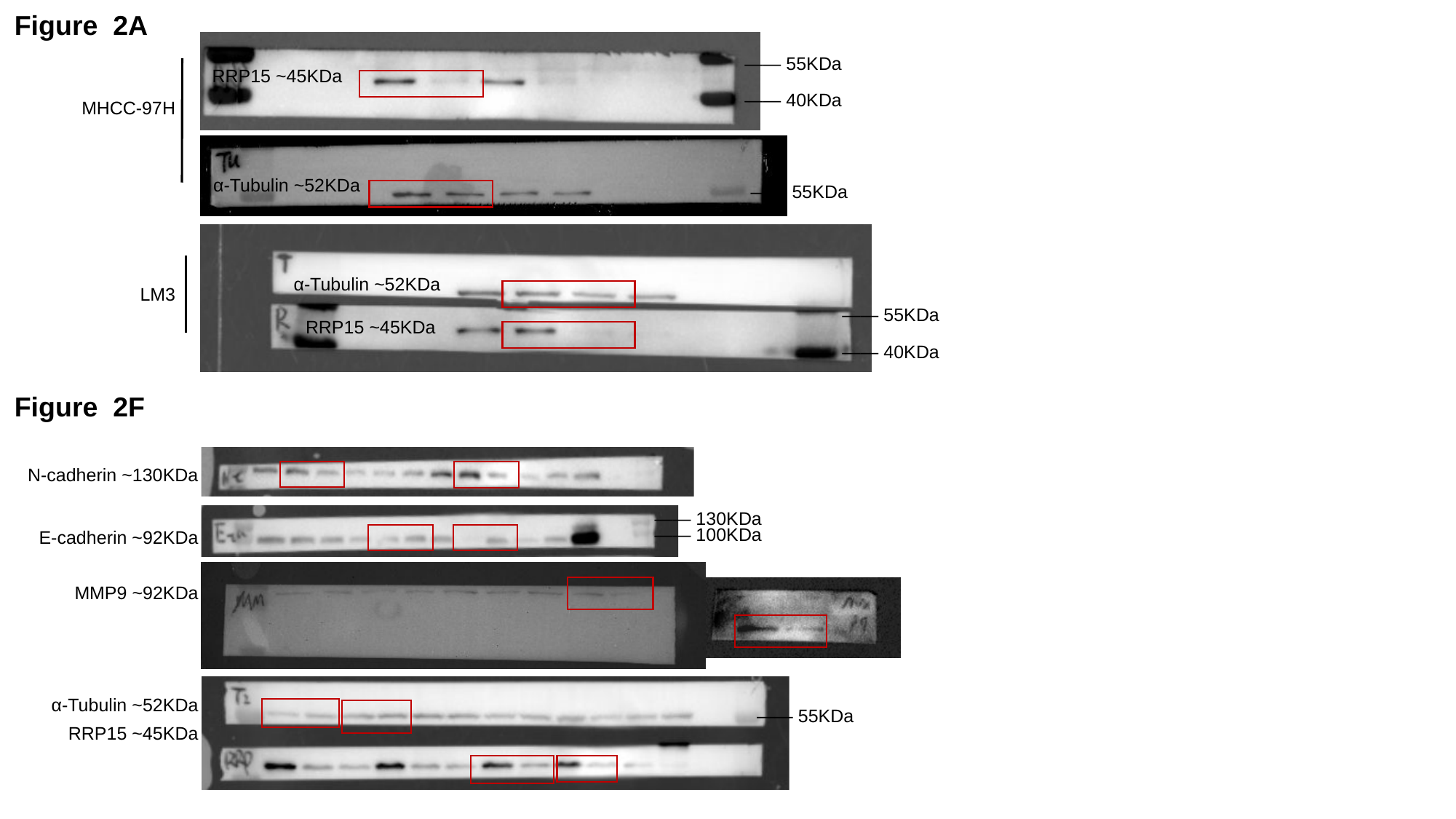

Figure 2A
—— 55KDa
RRP15 ~45KDa
—— 40KDa
MHCC-97H
α-Tubulin ~52KDa
—— 55KDa
α-Tubulin ~52KDa
LM3
—— 55KDa
RRP15 ~45KDa
—— 40KDa
Figure 2F
N-cadherin ~130KDa
—— 130KDa
—— 100KDa
E-cadherin ~92KDa
MMP9 ~92KDa
α-Tubulin ~52KDa
—— 55KDa
RRP15 ~45KDa

## Slide 3
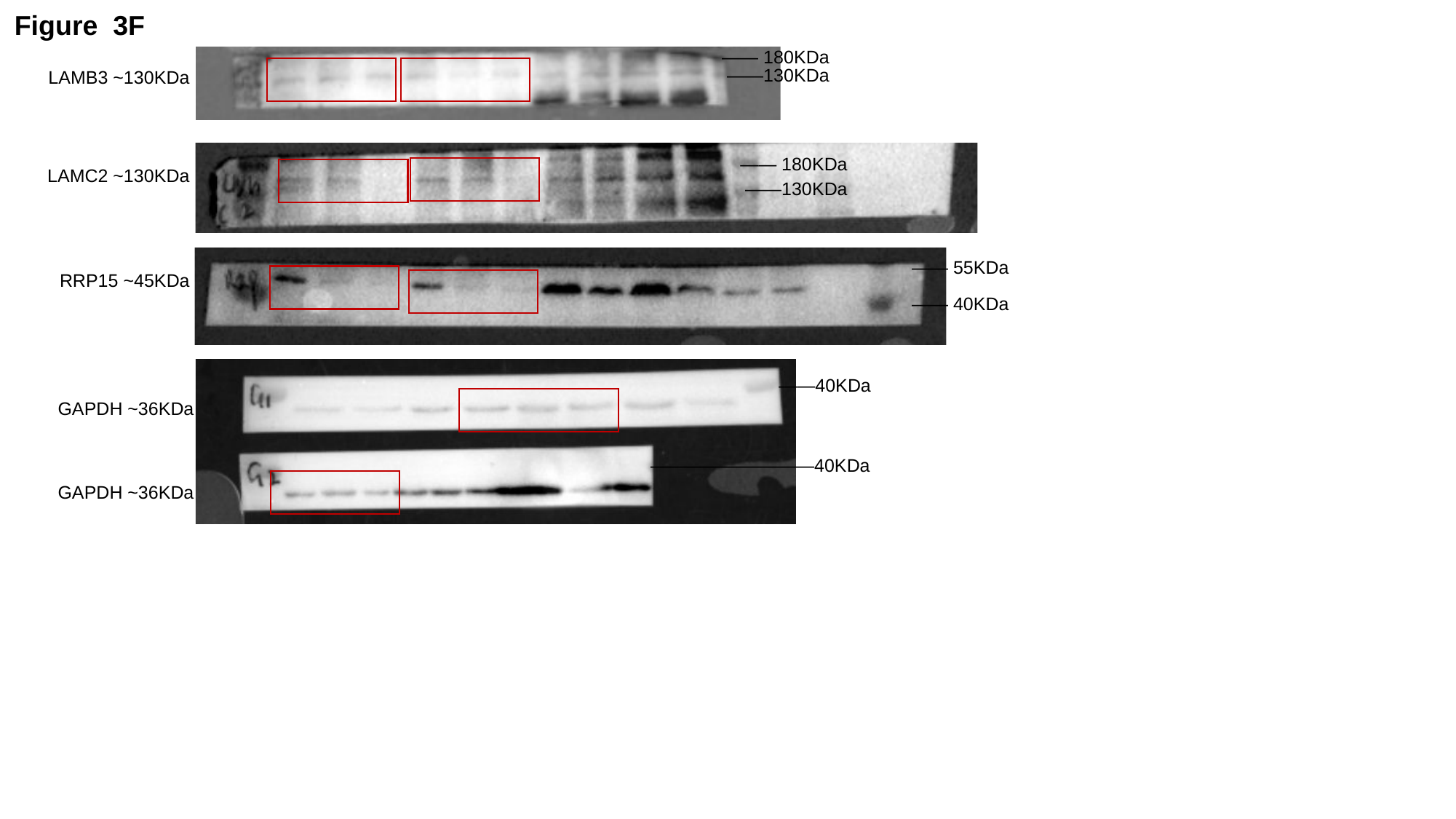

Figure 3F
—— 180KDa
——130KDa
LAMB3 ~130KDa
—— 180KDa
LAMC2 ~130KDa
——130KDa
—— 55KDa
RRP15 ~45KDa
—— 40KDa
——40KDa
GAPDH ~36KDa
—————————40KDa
GAPDH ~36KDa

## Slide 4
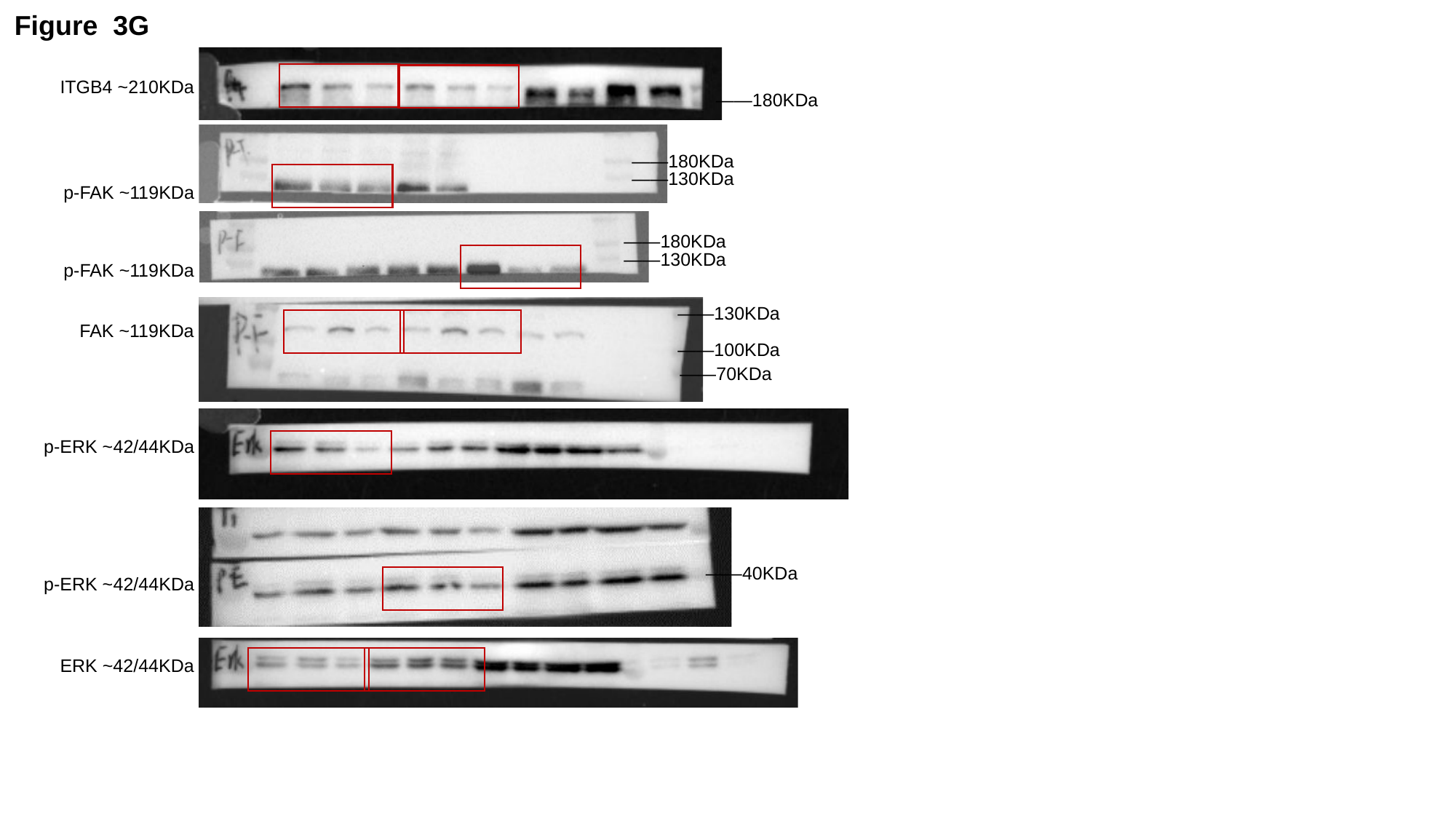

Figure 3G
ITGB4 ~210KDa
——180KDa
——180KDa
——130KDa
p-FAK ~119KDa
——180KDa
——130KDa
p-FAK ~119KDa
——130KDa
FAK ~119KDa
——100KDa
——70KDa
p-ERK ~42/44KDa
——40KDa
p-ERK ~42/44KDa
ERK ~42/44KDa

## Slide 5
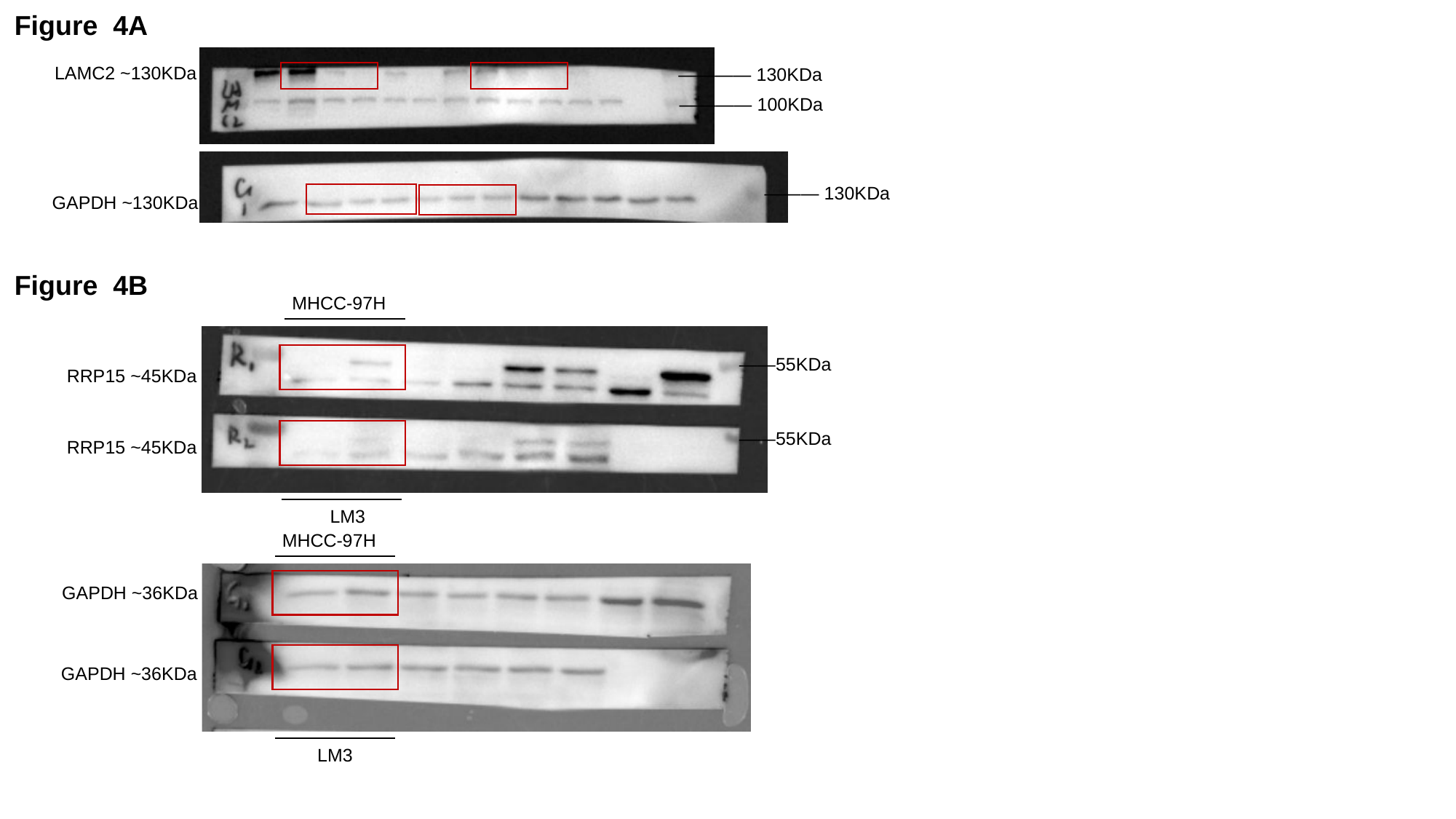

Figure 4A
LAMC2 ~130KDa
———— 130KDa
———— 100KDa
——— 130KDa
GAPDH ~130KDa
Figure 4B
MHCC-97H
——55KDa
RRP15 ~45KDa
——55KDa
RRP15 ~45KDa
LM3
MHCC-97H
GAPDH ~36KDa
GAPDH ~36KDa
LM3

## Slide 6
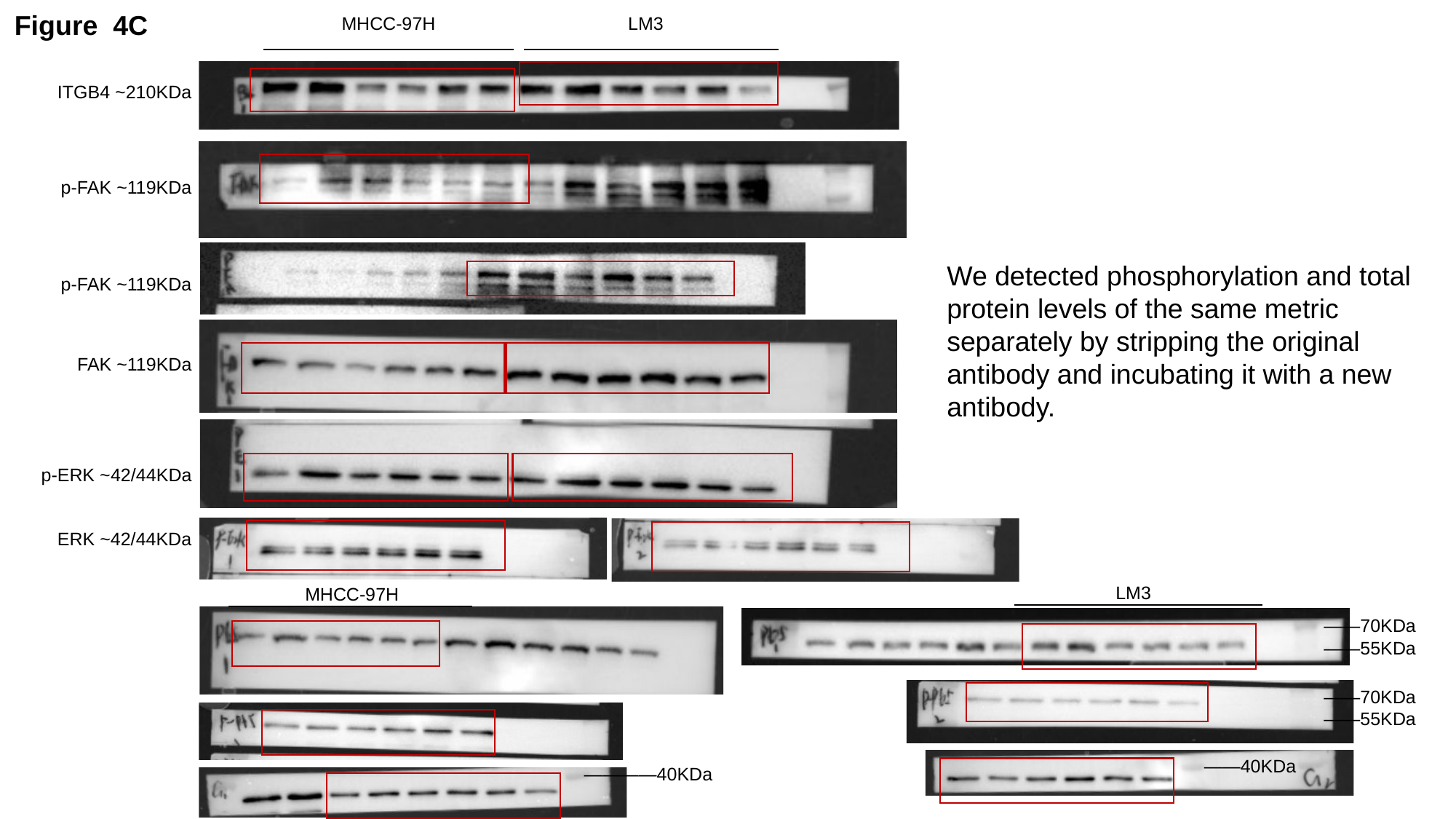

Figure 4C
MHCC-97H
LM3
ITGB4 ~210KDa
p-FAK ~119KDa
We detected phosphorylation and total protein levels of the same metric separately by stripping the original antibody and incubating it with a new antibody.
p-FAK ~119KDa
FAK ~119KDa
p-ERK ~42/44KDa
ERK ~42/44KDa
LM3
MHCC-97H
——70KDa
——55KDa
——70KDa
——55KDa
——40KDa
————40KDa

## Slide 7
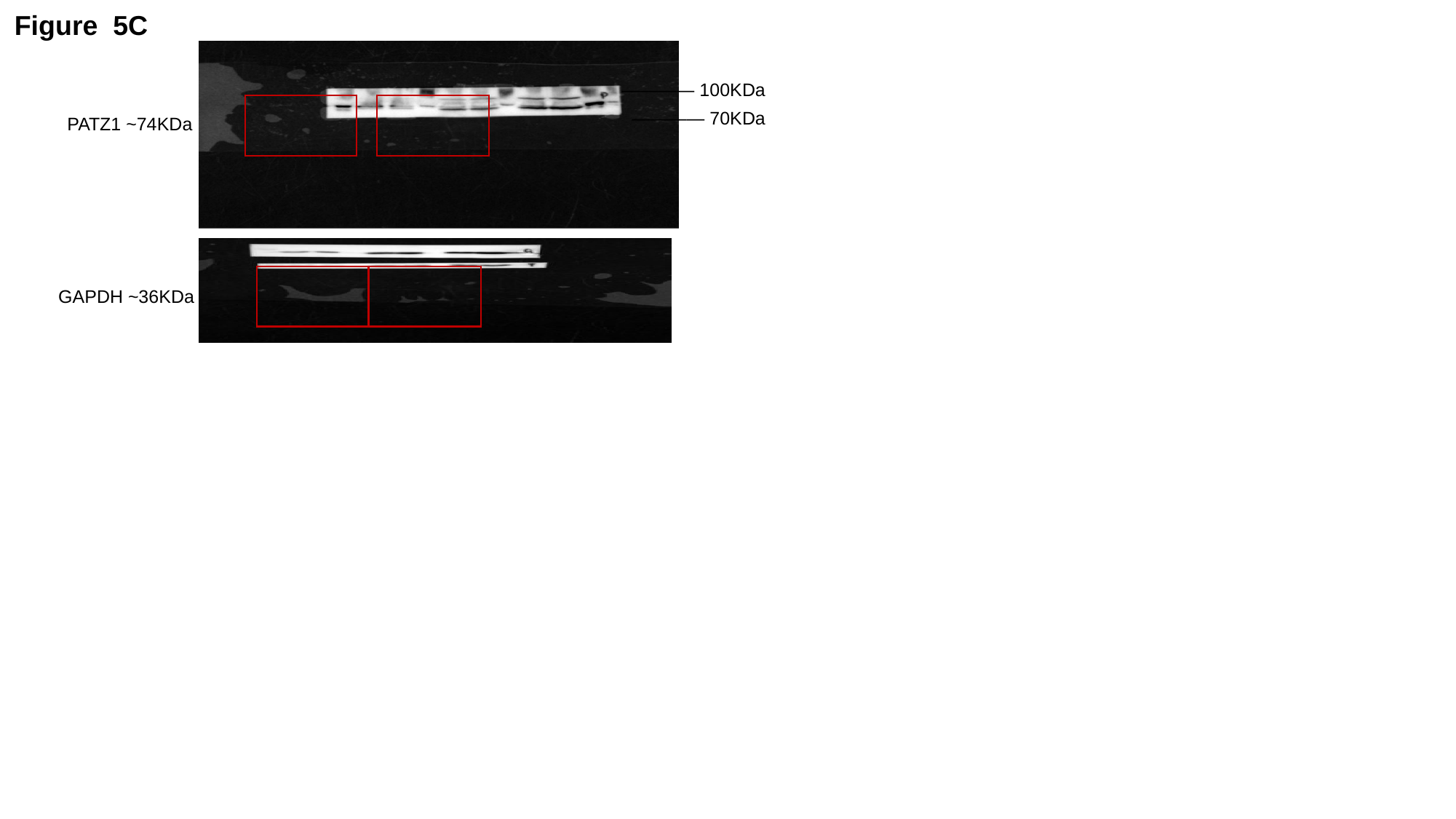

Figure 5C
———— 100KDa
———— 70KDa
PATZ1 ~74KDa
GAPDH ~36KDa

## Slide 8
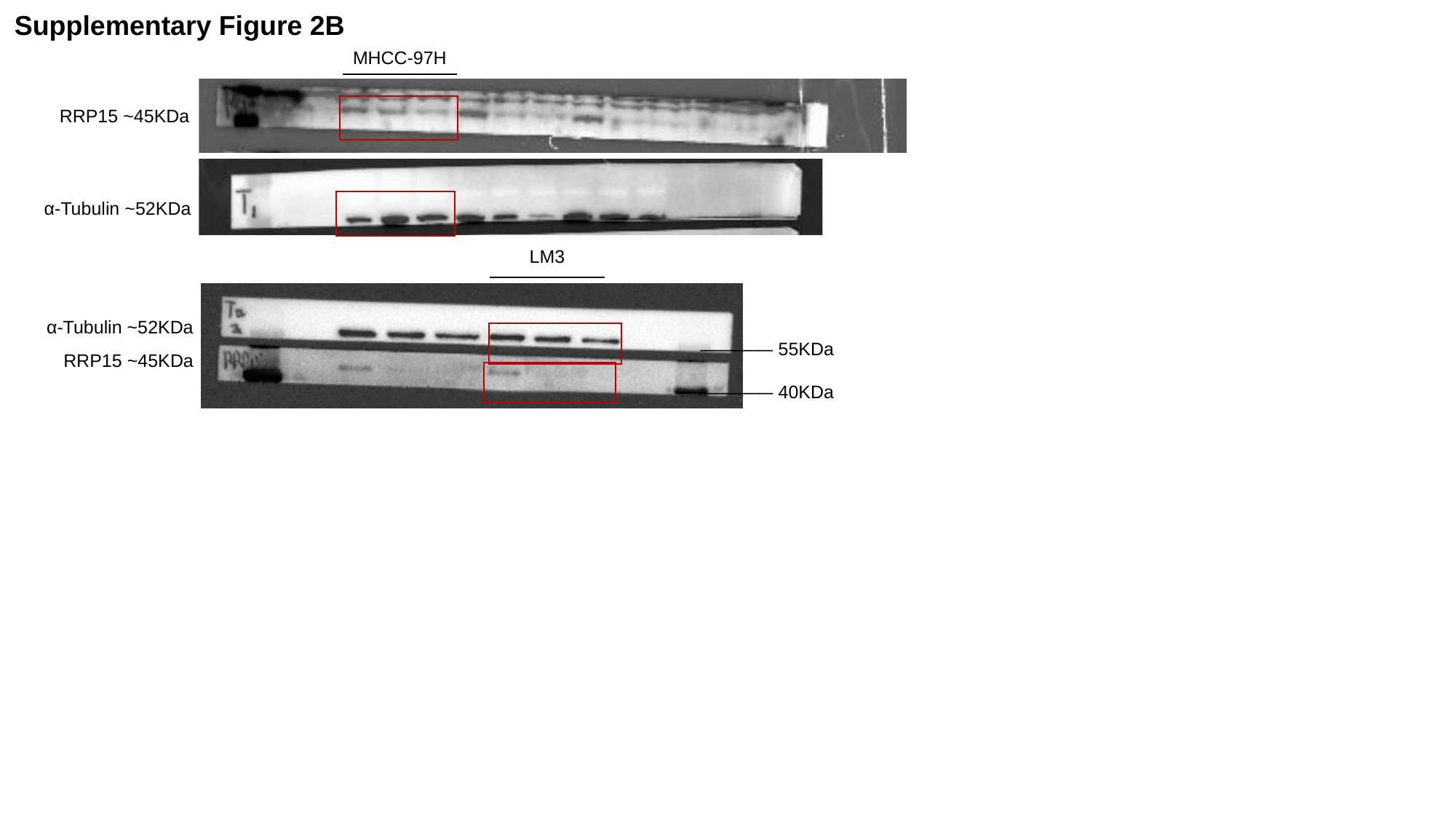

Supplementary Figure 2B
MHCC-97H
RRP15 ~45KDa
α-Tubulin ~52KDa
LM3
α-Tubulin ~52KDa
———— 55KDa
RRP15 ~45KDa
———— 40KDa

## Slide 9
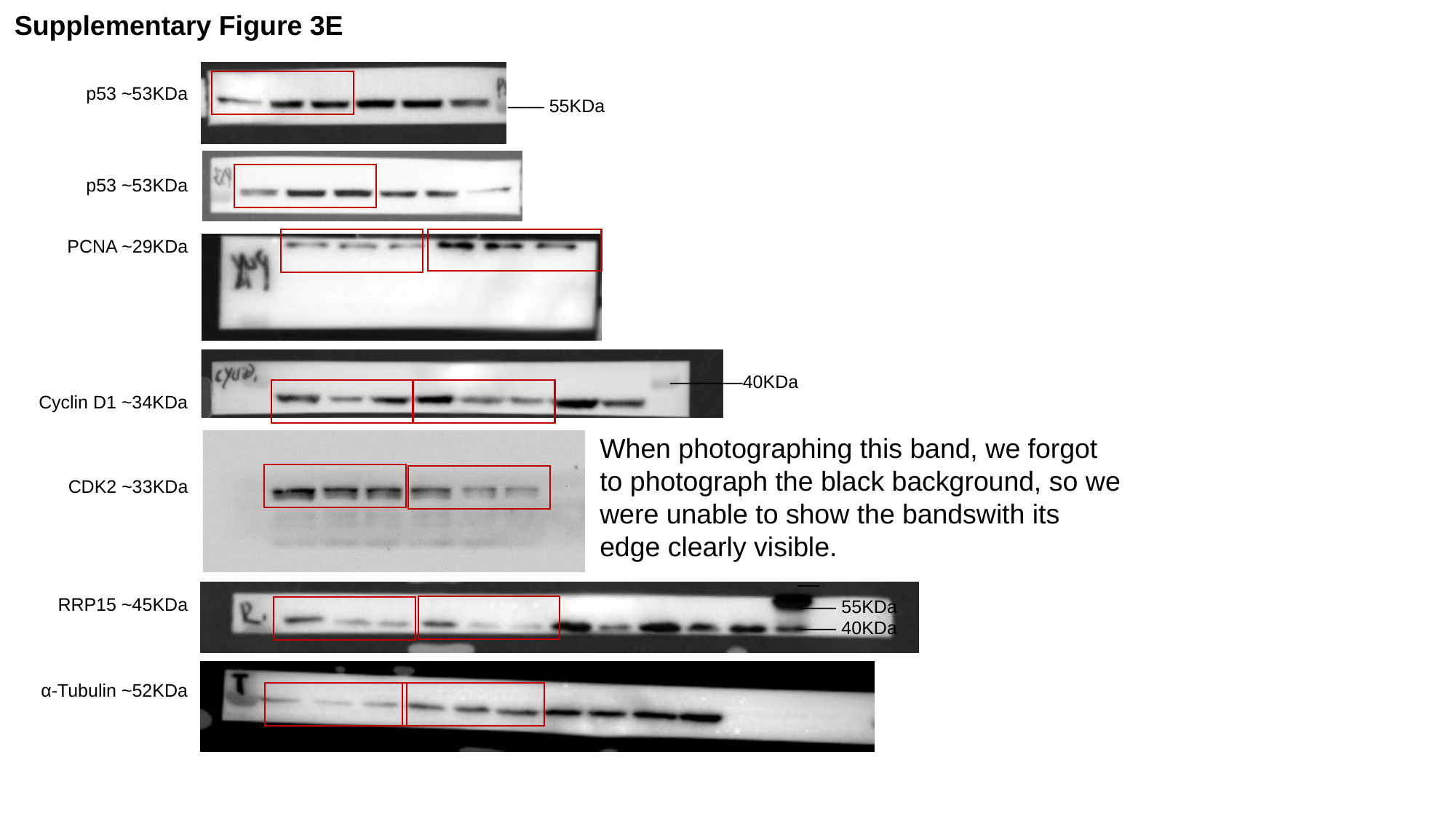

Supplementary Figure 3E
p53 ~53KDa
—— 55KDa
p53 ~53KDa
PCNA ~29KDa
————40KDa
Cyclin D1 ~34KDa
When photographing this band, we forgot to photograph the black background, so we were unable to show the bandswith its edge clearly visible.
CDK2 ~33KDa
RRP15 ~45KDa
—— 55KDa
—— 40KDa
α-Tubulin ~52KDa

## Slide 10
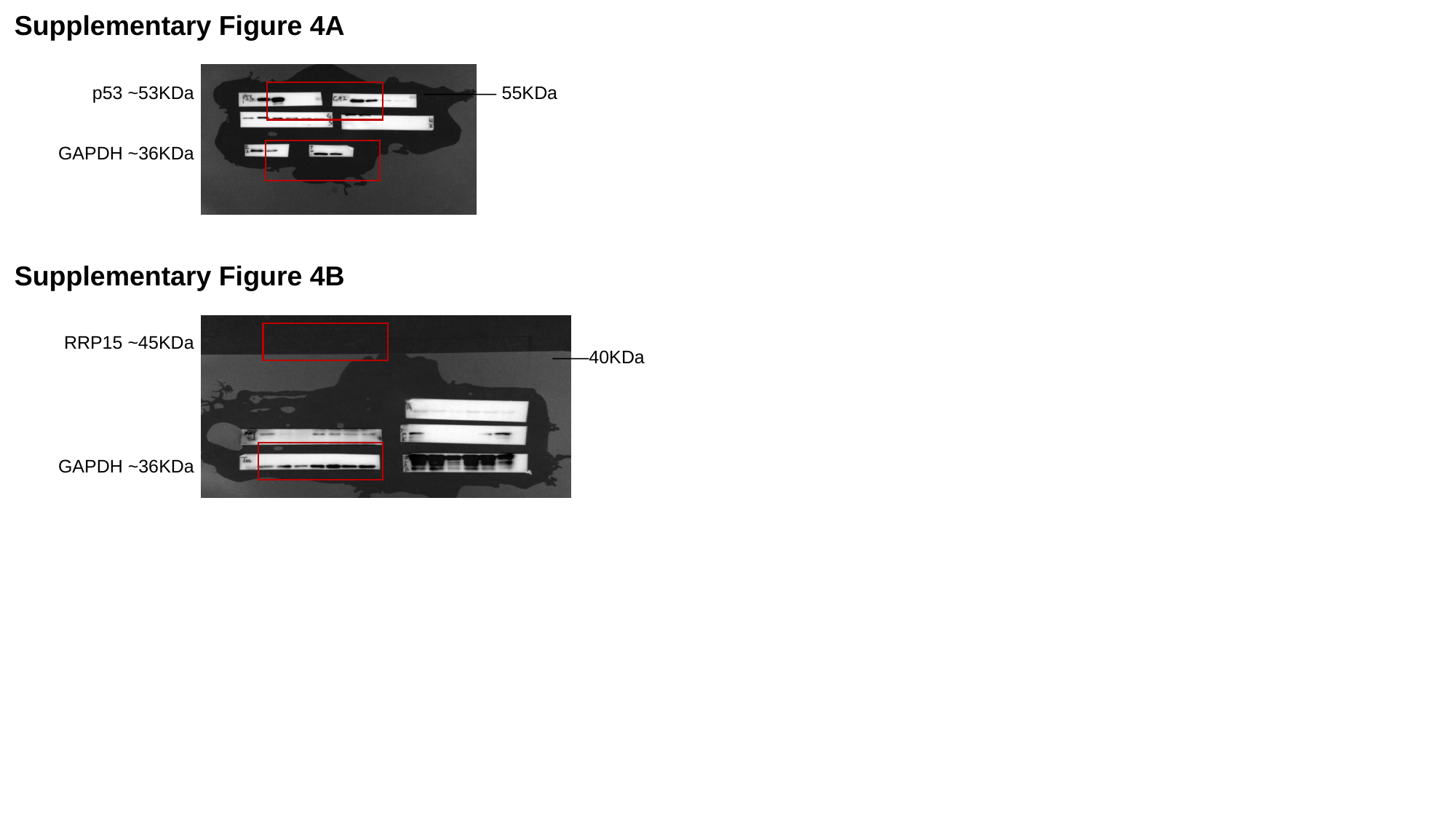

Supplementary Figure 4A
———— 55KDa
p53 ~53KDa
GAPDH ~36KDa
Supplementary Figure 4B
RRP15 ~45KDa
——40KDa
GAPDH ~36KDa
